# Woebot for Postpartum Mood and Anxiety: Randomized Controlled Trial Evaluating Feasibility, Acceptability, and Preliminary Efficacy of a Mobile Cognitive Behavioral Therapy Intervention

**DOI:** 10.2196/74083

**Published:** 2026-10-09

**Authors:** Toluwalase Ajayi, Jacqueline Kueper, Jill Waalen, Lauren Ariniello, Edward Ramos, Giulia Milan, Katie Baca-Motes, Andre Williams, Lauren Bullard, Athena Robinson

**Affiliations:** 1 University of California, San Diego, 9300 Campus Point Drive, MC 7196, La Jolla, CA, 92037, United States, 1 7852181643; 2 Scripps Research Institute, La Jolla, CA, United States; 3 Care Evolution, Ann Arbor, MI, United States; 4 Woebot Health, San Francisco, CA, United States

**Keywords:** postpartum mental health, postpartum depression, health equity, digital health, mobile apps, relational agent, chatbot, cognitive behavioral therapy, mobile phone

## Abstract

**Background:**

Postpartum psychological distress, ranging from transient mood and anxiety disturbances to full-syndrome postpartum depression, is prevalent. Many postpartum individuals lack access to evidence-based interventions due to stigma and insufficient provider availability. The treatment gap is particularly pronounced among historically marginalized groups, including Black, Hispanic/Latina, and low-income mothers. Digital health interventions offer support to address this.

**Objective:**

We aimed to evaluate the feasibility, acceptability, and preliminary efficacy of Woebot for Postpartum Mood and Anxiety (W-PPMA), a smartphone-based relational agent delivering cognitive behavioral therapy–informed psychoeducation to postpartum individuals.

**Methods:**

This randomized controlled trial recruited participants from the PowerMom study, a digital platform for maternal health research. Eligible individuals (≥16 y, <3 mo post partum) were randomized to W-PPMA or a waitlist control condition. W-PPMA, an investigational digital mental health intervention, features a relational agent delivering cognitive behavioral therapy–based psychoeducation via text-based conversations. Primary outcomes included feasibility (Usage Rating Profile–Intervention Revised, Feasibility Subscale), acceptability (Usage Rating Profile–Intervention Revised, Acceptability Subscale), and satisfaction (Client Satisfaction Questionnaire–8) assessed at the 8-week end of intervention (EOI). The secondary outcome was change in depressive symptoms (Patient Health Questionnaire–8) among those with elevated baseline symptoms. Exploratory outcomes included anxiety (Generalized Anxiety Disorder–7 items), stress (Perceived Stress Scale), perinatal depression (Edinburgh Postnatal Depression Scale), Mother-to-Infant Bonding Scale, and therapeutic alliance (Working Alliance Inventory–Short Revised). This study followed CONSORT (Consolidated Standards of Reporting Trials) guidelines and received Institutional Review Board approval.

**Results:**

Of 267 participants (W-PPMA=144, waitlist=123), mean age was 32.2 (SD 5.1) years; 12.7% (n=34) identified as Hispanic/Latina, 8.6% (n=23) as Black, and 68.5% (n=183) as White. W-PPMA users engaged with the app a median of 9 (IQR 5‐24) days across 4 active weeks (IQR 2‐7). At EOI, W-PPMA participants reported high feasibility (Usage Rating Profile–Intervention Revised, Feasibility Subscale median 31, IQR 28‐34), acceptability (Usage Rating Profile–Intervention Revised, Acceptability Subscale median 30, IQR 28‐34), and satisfaction (Client Satisfaction Questionnaire–8 median 26, IQR 24‐29). Among those with baseline depressive symptoms (n=97), Patient Health Questionnaire–8 scores improved modestly in the W-PPMA group compared to waitlist (mean change −0.7, SD 3.4, vs 0.0, SD 4.8; Cohen *d*=−0.16). Exploratory analyses showed a statistically significant between-group difference in Perceived Stress Scale change at EOI (mean difference −1.59, 95% CI −2.4 to −0.73) and a nonsignificant between-group difference in Edinburgh Postnatal Depression Scale change (mean difference −0.51, 95% CI −1.36 to 0.34). Therapeutic alliance (Working Alliance Inventory–Short Revised) was strongest at baseline among Black participants and those from socioeconomically disadvantaged neighborhoods (Area Deprivation Index ≥75). Participants with a high school or General Educational Development reported the highest satisfaction scores, underscoring accessibility across educational backgrounds.

**Conclusions:**

W-PPMA met primary study aims. Preliminary evidence and these findings support W-PPMA, warranting further evaluation in larger trials.

## Introduction

### Prevalence and Burden of Postpartum Blues and Postpartum Depression

The postpartum period is a time of significant lifestyle, relational, and physical health changes, often accompanied by psychological distress and profound impacts on maternal mood. In this study, we use the term postpartum “psychological distress” to refer to a spectrum ranging from transient mood and anxiety disturbances to full-syndrome postpartum depression (PPD), consistent with the most common manifestations in the early postpartum period. Broader definitions of perinatal mood and anxiety disorders also include posttraumatic stress symptoms [1], which were not assessed in this study. Understandably, many mothers experience various mood and anxiety symptoms and labile emotions after birth, such as mood swings, crying spells, anxiety, irritability, sadness, and feelings of being overwhelmed [2-4]. These symptoms, often referred to as “baby blues,” typically begin within the first 2‐3 days and resolve without intervention within 2 weeks for most individuals [5]. However, recognition and management of persistent postpartum mood and anxiety symptoms is critical, as they are not only distressing to mothers and their families but also represent a risk factor for the development of full-syndrome PPD and anxiety disorders [4,6].

As many as 75% of women report some degree of mood and/or anxiety symptoms after birth [5]. Full-syndrome PPD affects approximately 13%‐20% of postpartum individuals in the United States, with prevalence varying significantly by state and demographic group [4,7]. PPD is associated with adverse outcomes, including increased maternal morbidity and mortality [8], impaired maternal-child interactions [9], diminished parenting behaviors, and cognitive, emotional, and behavioral challenges in child development [10].

### Current Treatment

Treatment of peripartum mood and anxiety symptoms typically involves psychotherapy and/or antidepressant therapy. Cognitive behavioral therapy (CBT) and interpersonal psychotherapy (IPT) are recommended as first-line treatments for mild to moderate postpartum mood and anxiety concerns, while antidepressant pharmacotherapy is reserved for more severe cases of PPD [11]. CBT aims to address the reciprocal interactions between thoughts, feelings, and behaviors by challenging negative thought patterns and promoting adaptive behaviors [12]. IPT focuses on alleviating symptoms by improving interpersonal functioning, communication, and role clarification in response to relational challenges [13]. Techniques derived from these psychotherapies are beneficial not only for mothers diagnosed with PPD but may also provide effective mental health and emotional support for nonclinical populations.

### Barriers to Mental Health Care Support

Despite the existence of evidence-based treatments for peripartum mood and anxiety, many postpartum individuals do not seek or engage in care, even when recommended by health care providers [14,15]. Stigma surrounding mental health is a significant barrier to help-seeking and treatment usage [16]. Additional barriers include a shortage of qualified therapists, inflexible scheduling, long wait times, childcare challenges, transportation difficulties, and affordability concerns [14]. For pharmacotherapy, concerns about antidepressant transmission through breast milk further limit its use [17]. These barriers are especially pronounced among Black, Hispanic/Latina, and low-income mothers, who face higher risks of PPD and significant systemic inequities in accessing care [18].

Low representation of historically marginalized groups in health research exacerbates disparities, reducing the generalizability of findings and hindering the development of effective interventions [19,20]. Ensuring diverse inclusion in research is critical to addressing health inequities and improving outcomes.

### Digital Interventions

Digital mental health interventions (DMHIs) offer promise for postpartum mental health support. In this paper, we focus on fully automated, app-based DMHIs such as Woebot, which deliver psychoeducation and coping strategies without requiring a live clinician. We acknowledge that telemedicine-delivered psychotherapy, such as CBT provided by a therapist via video or phone, has also grown substantially, with recent evidence [21] showing it to be noninferior to in-person care with high satisfaction rates across diverse populations. While this is an important parallel development, our study centers on the potential of automated DMHIs to scale support where clinician-delivered therapy is not available [22]. Such DMHIs provide private, stigma-free support, which is particularly relevant for postpartum mothers navigating the unique demands of early parenthood [23]. Moreover, their scalability enables broader population reach, including individuals who might otherwise receive no care.

To address these challenges, this study leveraged the PowerMom mobile research platform [24], chosen for its ability to facilitate decentralized, bilingual (English and Spanish), and scalable data collection for maternal health. PowerMom’s capacity to enable remote participation, inclusivity, and generalizability makes it valuable for engaging underrepresented populations in research.

The intervention evaluated in this study is Woebot for Postpartum Mood and Anxiety (W-PPMA), an investigational DMHI that offers a guided self-help program that delivers CBT psychoeducation and self-management techniques through brief text-based conversations with a relational agent named Woebot. Woebot is intended to present to users a friendly, helpful, self-help ally that is explicitly not a human or a therapist. Neither W-PPMA nor Woebot have been evaluated, cleared, or approved by the Food and Drug Administration and are not available for general use. W-PPMA integrates evidence-supported techniques from CBT and IPT, specifically tailored to the postpartum experience. Earlier prototypes of Woebot have demonstrated feasibility, acceptability, and preliminary efficacy in improving anxiety and depression symptoms among young adults [25], postpartum women [26,27], and adults addressing substance use [28,29]. Building on these foundations, W-PPMA aims to offer a specialized approach to address the unique mental health needs of postpartum mothers. While prior work in postpartum populations has been limited to smaller pilot trials, this study extends this evidence by conducting the largest randomized evaluation of Woebot in a postpartum cohort to date, with a deliberate emphasis on recruiting underrepresented racial, ethnic, and socioeconomic groups.

The primary aim of this study is to evaluate the feasibility and acceptability of W-PPMA, using app usage and validated measures, among a diverse group of postpartum women. The secondary aim is to assess the preliminary efficacy of W-PPMA relative to a waitlist control condition in managing symptoms of depression at the 8-week end of intervention (EOI) among participants with elevated baseline depressive symptoms. Exploratory aims include (1) examining potential between-group differences in perceived mother-infant bonding, mood and anxiety symptoms, and perceived stress; (2) evaluating therapeutic bond with Woebot at 1 and 8 weeks; and (3) assessing feasibility, acceptability, perceptions of stigma as a barrier to mental health care, and mood outcomes across key sociodemographic variables (eg, race and ethnicity, age, education, gender, marital status, health insurance, and employment status) at baseline and 8 weeks.

Despite the significant prevalence and burden of postpartum psychological distress, existing interventions remain underused, particularly among historically marginalized groups who face systemic barriers to care. While evidence-based psychotherapies such as CBT and IPT are effective in treating postpartum mood disorders, there is a critical need to explore how these interventions can be feasibly and equitably delivered to diverse populations in nonclinical settings. Digital health tools, such as mobile applications, hold immense promise for addressing these gaps by overcoming logistical and stigma-related barriers. However, few digital interventions have been rigorously studied in postpartum populations. By evaluating the feasibility, acceptability, and preliminary efficacy of W-PPMA in a diverse and bilingual cohort, this study aims to advance understanding of scalable, accessible mental health solutions. Ultimately, this research provides critical insights into closing the postpartum mental health treatment gap with an emphasis on equity and inclusion.

## Methods

### Ethical Considerations

This study was approved as human participants research by the Institutional Review Board (IRB) of the Scripps Research Translational Institute (IRB-21‐7738). This study was conducted at the Scripps Research Translational Institute. All participants provided informed e-consent before participation. Deidentified data were transferred to the Scripps Research Translational Institute for analysis.

### Study Design

This randomized waitlist-controlled trial evaluated the W-PPMA digital health tool. The trial consisted of 3 phases: screening/baseline, treatment, and follow-up. Participants were assessed at screening/baseline, day 3, week 4, week 8 (EOI), week 12, and week 16 (end of study [EOS]). The day 3 assessment was included to capture early usability and engagement signals, as well as to verify baseline stability immediately post randomization. All primary end points were evaluated at week 8 (EOI). A waitlist control design was selected to balance methodological rigor with ethical considerations. This approach allowed all participants eventual access to W-PPMA after the 8-week EOI assessment, thereby minimizing the risk of withholding potentially beneficial support from a vulnerable postpartum population. The design was reviewed and approved by the IRB as consistent with ethical standards for studies involving mental health interventions.

### Recruitment Strategy

Pregnant and postpartum participants were recruited through the PowerMom baseline research study (Scripps Research Digital Trials Center), a mobile app-based research initiative hosted on the HIPAA (Health Insurance Portability and Accountability Act)–compliant MyDataHelps (CareEvolution, LLC) clinical research platform. PowerMom enables participants to access prenatal health resources while contributing pregnancy-related data to research. Recruitment for PowerMom was supported by a bilingual, multichannel campaign (eg, email, social media, and in-app nudges) involving PowerMom Consortium [30] partners from health and technology organizations, maternal advocacy groups, and community health centers. Key partners included Microsoft, Google/Fitbit, Philips, March of Dimes, and others, and community-based organizations serving Black and Hispanic/Latina populations. Recruitment campaigns outside of consortium partners also included Ovia Health (Labcorp) platform.

English-speaking PowerMom participants received information about the Woebot substudy through email and in-app notifications. Interested PowerMom participants who were in the postpartum period were directed to the Woebot eligibility screening, which included a disclaimer about the sensitive nature of some questions. Eligible participants provided informed consent for the Woebot substudy, and participation in the Woebot substudy did not affect their involvement in the PowerMom baseline study.

The recruitment period for the Woebot substudy was from February 2023 to January 2024. The initial goal was to recruit 225 participants in each arm of this study. To promote inclusivity, recruitment aimed to enroll at least 30% of participants from underrepresented racial and ethnic groups and 50% from groups underrepresented in biomedical research (UBRs), as defined by the NIH (National Institutes of Health) All of Us Research Program [31]. UBR criteria included racial/ethnic minorities, low socioeconomic status, rural residency, and other categories. To achieve this distribution, the UBR criteria were built into the enrollment workflow in this study’s app. This study’s app was configured such that enrollment would be paused for any non-UBR participant until we had reached 225 enrolled and consented participants who self-identify from a UBR race or ethnicity or other UBR category. A study coordinator actively monitored enrollment numbers to ensure recruitment targets were being met.

However, the sponsor nominated to end enrollment early (after n=296 consented) given that the a priori goal of sample diversity (50% UBR) was successfully achieved early and also to advance time to results given the importance and relevance of the hypotheses tested herein. The original sample size target (n=450) was selected to provide approximately 80% power to detect a small-to-moderate effect size (Cohen *d* ≈0.30) in change in depressive symptoms (Patient Health Questionnaire–8 [PHQ-8]) at α=.05. Given early closure of enrollment, the final analytic sample (N=267) was underpowered for efficacy analyses, and these outcomes are presented as exploratory.

### Inclusion and Exclusion Criteria

Eligible participants were PowerMom enrollees aged 16 years or older, less than 3 months postpartum, English-literate, smartphone users, and willing to engage with the program and assessments for 16 weeks.

Exclusion criteria included a lifetime diagnosis of psychotic or bipolar disorders, suicidal ideation or attempts within the past 12 months, substance use within the past 12 months, pregnancy loss in the past 18 months, or prior use of Woebot programs.

### Procedures

Participation spanned 16 weeks, including an 8-week intervention or waitlist control period, followed by exploratory assessments at 12- and 16-weeks. After completing baseline assessments, participants were randomized 1:1 into the W-PPMA group or the waitlist control group. Neither participants nor staff were blinded to group assignments.

Participants randomized to W-PPMA accessed the app from randomization through 16 weeks (EOS) and were encouraged to use it daily, with a recommended minimum of 5 minutes per day during the treatment phase. Waitlist participants accessed W-PPMA starting at 8 weeks (EOI). Assessments were conducted via the PowerMom app at baseline, day 3, 4-week, 8-week (EOI), 12-week, and 16-week (EOS) time points. Each assessment took 10‐15 minutes, and participants received reminders via email and PowerMom app push notifications.

Participants were eligible to receive up to US $175 in Amazon e-gift cards as compensation, prorated across completion of scheduled study assessments (eg, baseline, midintervention, EOI, and follow-up surveys). The compensation schedule was designed to acknowledge participant time burden across multiple assessments and to support retention over the 16-week study period while remaining modest to minimize undue inducement; the amount and structure were reviewed and approved by the IRB and were consistent across all participants to ensure fairness and transparency. Gift cards were distributed electronically after the completion of each assessment time point. During enrollment, fraudulent activity was detected, characterized by anomalous enrollment patterns (eg, implausibly rapid e-consent and survey completion times and duplicate IP addresses) and an unusual and rapid spike in enrollment across both the baseline study and the Woebot substudy. A small number of fraudulent accounts received e-gift card links before being identified. To ensure data integrity, all fraudulent enrollments were excluded before randomization, and participants who failed to complete baseline assessments were classified as screen failures and not included in this study. Only verified participants who met all eligibility criteria and completed baseline assessments were randomized. The final analytic sample (N=267) excluded all fraudulent accounts and therefore represents postpartum individuals meeting the intended inclusion criteria.

### Intervention Groups

#### W-PPMA

W-PPMA is an investigational DMHI that offers an 8-week guided self-help program that combines CBT- and IPT-based techniques tailored to provide mental health support to postpartum women. Through a text-based interface, a relational agent named Woebot engages users in conversations and provides users with self-guided psychoeducation and mood and anxiety management strategies. The program includes daily push notifications to reengage users and encourage consistent participation. Neither W-PPMA nor Woebot have been evaluated, cleared, or approved by the Food and Drug Administration and are not available for general use.

#### Waitlist Control

Waitlist control participants had access to W-PPMA from the 8-week (EOI) through to the 16-week (EOS) period with similar usage recommendations.

### Safety Considerations

W-PPMA followed safety recommendations from the American Psychiatric Association [32] and American Medical Association [33]. W-PPMA has a language detection protocol that is based on a natural language processing algorithm designed to detect if a user inputs, via free-response text entry, concerning phrases or words that match an a priori identified and thorough list that may be used in crises. Upon detection of any concerning language, the language detection protocol reminds the participant of the app’s limitations of services—which are stated in the study’s informed consent, the W-PPMA mobile app’s privacy policy and terms of service, as well as the intervention’s onboarding screens—and offers a resource list of readily accessible support channels, including emergency contact phone numbers and suicide ideation and domestic violence hotline information.

Additionally, in the event that a participant endorses having current suicidal ideation or a plan when answering the screening questions, an automatic email would be sent to this study’s team. Study personnel would then call 911, notify the operator that it is a psychiatric emergency, and provide a trained emergency response person with the participant’s phone number to follow up with them. All participants were informed of these procedures in this study’s consent form, which explicitly outlined the protocol for responding to safety concerns, including emergency escalation and study personnel involvement in crises.

### Assessments

Baseline assessments collected demographic data, including age, race, ethnicity, gender identity, marital status, education, employment, and insurance status. Neighborhood socioeconomic disadvantage was assessed using the Area Deprivation Index (ADI) from the Neighborhood Atlas, which ranks census block groups nationwide by socioeconomic disadvantage, with higher scores indicating greater disadvantage. Pregnancy history included the number of prior pregnancies, outcomes, and any fetal demise within the exclusionary period. Psychiatric history assessed diagnosis of psychotic disorders, mood disorders, and suicidality within the past 12 months, and substance use during the same period. Current or prior use of psychiatric medications and therapy was also documented.

### Primary Outcomes: Feasibility, Acceptability, and Satisfaction Measure

Regarding Usage Rating Profile–Intervention Revised (URP-IR) [34], the 6-item feasibility (Usage Rating Profile–Intervention Revised, Feasibility Subscale [URPI-F]) and 6-item acceptability (Usage Rating Profile–Intervention Revised, Acceptability Subscale [URPI-A]) subscales were used to measure feasibility (eg, “The total time required to do the treatment procedures was manageable”) and acceptability (eg, “I liked the procedures used in this treatment”). Responses ranged from 1 (“strongly disagree”) to 6 (“strongly agree”), with total scores ranging from 6 to 36 and higher average scores indicating greater feasibility or acceptability. The URP-IR demonstrates strong reliability, internal consistency, and discriminant validity [34].

Regarding Client Satisfaction Questionnaire (CSQ-8) [35], this widely used 8-item measure evaluated participants’ satisfaction with W-PPMA on a 4-point scale (eg, “How would you rate the quality of service you received?”). Total scores ranged from 8 to 32, with higher scores indicating greater satisfaction.

Regarding app engagement, engagement with the W-PPMA app was assessed using quantitative metrics, including the number of active days, messages sent per week, and the number of content modules completed.

For interpretability, thresholds were applied to define high feasibility, acceptability, and satisfaction. On the URPI-F and URPI-A subscales (range 6‐36), scores ≥30 were considered indicative of strong feasibility or acceptability. On the CSQ-8 (range 8‐32), scores ≥25 were considered indicative of high satisfaction. These thresholds are consistent with prior applications of these validated instruments.

### Secondary Outcome: Symptoms of Depression

The PHQ-8 is an 8-item measure evaluating depressive symptom severity over the past 2 weeks [36]. Scores range from 0 to 24, with cut points of 5, 10, 15, and 20 corresponding to mild, moderate, moderately severe, and severe depression, respectively [37,38]. The PHQ-8 is a reliable and valid tool for screening depressive symptoms [39].

### Exploratory Outcomes

The Edinburgh Postnatal Depression Scale (EPDS) is a 10-item self-report tool that screens for postnatal depression [40]. For this study, the suicidality item was omitted; therefore, 9 items were administered and the total score ranged from 0 to 27, with higher scores indicating greater depressive symptom severity. The EPDS is widely validated for postpartum populations [41]. Both the PHQ-8 and EPDS were included to capture depressive symptoms: the PHQ-8 is widely validated for general populations and aligned with our primary depression outcome, while the EPDS is specific to perinatal populations and allows comparability with prior postpartum research.

Regarding Generalized Anxiety Disorder (GAD-7), the GAD-7 is a 7-item measure assessing anxiety symptoms over the past 2 weeks [42]. Scores range from 0 to 21, with cut points of 5, 10, and 15 indicating mild, moderate, and severe anxiety, respectively. This measure has demonstrated high reliability and validity [43].

Regarding Perceived Stress Scale–10, this 10-item scale measures perceived stress in the past month [44]. Responses range from 0 (“never”) to 4 (“very often”) and scores range between 0 and 40, with higher scores indicating greater perceived stress. The Perceived Stress Scale–10 demonstrates strong reliability and validity [45].

Regarding the Mother-to-Infant Bonding Scale (MIBS), the MIBS includes 8 one-word emotional descriptors (eg, “loving” or “resentful”) rated on a 4-point scale from 0 (“very much”) to 3 (“not at all”). Total scores range from 0 to 24, with lower scores indicating stronger mother-to-infant bonding [46,47].

Regarding the Working Alliance Inventory–Short Revised (WAI-SR), the 12-item WAI-SR measures therapeutic alliance using 3 subscales: task agreement, goal agreement, and affective bond [48]. Items were adapted to refer to Woebot as the therapist. Responses range from 1 (“never”) to 5 (“always”), with higher scores indicating a stronger therapeutic alliance.

Regarding Perceived Barriers to Psychological Treatment (PBPT), the PBPT [49] identifies potential barriers to engaging in mental health treatment. This study used the 7-item Stigma subscale (eg, “My concern about being judged makes it ____ for me to attend counseling”), with responses ranging from 1 (“not difficult at all”) to 5 (“impossible”).

Figure 1 presents the schedule of assessments by study group.

**Figure 1.**
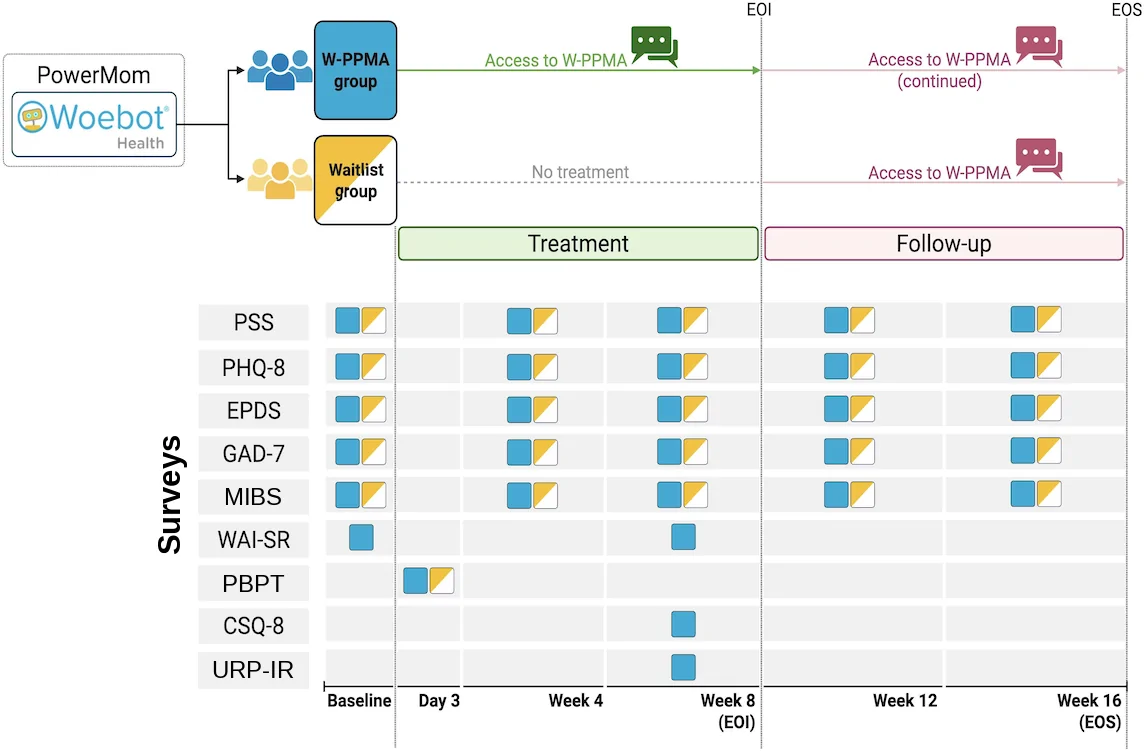
Schedule of assessment. Time point where each outcome measure was administered and whether this was for all participants or only those in the W-PPMA group. Week 8 represents EOI, after which the waitlist control group additionally gained access to the Woebot app. CSQ-8: Client Satisfaction Questionnaire-8 items; EOI: end of intervention; EOS: end of study; EPDS: Edinburgh Postnatal Depression Scale; GAD-7: Generalized Anxiety Disorder Scale; MIBS: Mother-to-Infant Bonding Scale; PBPT: Perceived Barriers to Psychological Treatment, Stigma Subscale; PHQ-8: Patient Health Questionnaire–8; PSS: Perceived Stress Scale; URP-IR: Usage Rating Profile–Intervention Revised; W-PPMA: Woebot for Postpartum Mood and Anxiety; WAI-SR: Working Alliance Inventory–Short Revised.

### Statistical Analysis

Primary analyses were conducted using an intention-to-treat strategy whereby participants were analyzed in the treatment group to which they were assigned. Per protocol (PP) sensitivity analyses were performed for the CSQ-8, URPI-A, URPI-F, PHQ-8, GAD-7, EPDS, Perceived Stress Scale (PSS), MIBS, and WAI-SR outcome measures. These sensitivity analyses excluded participants who deviated from the protocol in one or more of these ways: relevant measures not completed in full within the preplanned timing window, withdrew from this study, and, if in the W-PPMA group, did not open the app at least once per week for at least half of the intervention weeks. The latter criterion was based on previous research into app usage patterns.

Sample characteristics, including participant self-reported sociodemographic, psychiatric, and pregnancy history, and prior and concomitant medications and therapy characteristics, were described overall and by intention-to-treat study group.

The primary outcomes of feasibility, acceptability, and satisfaction were described for the W-PPMA study group at the 8-week time point (EOI).

The secondary outcome to assess the preliminary efficacy of W-PPMA relative to a waitlist control condition to manage symptoms of depression was analyzed among the subgroup of participants with PHQ-8 greater than or equal to 5 at baseline (minimal to several symptoms of depression). Mean 8-week change scores were calculated and compared using a 2-sided independent *t* test. Cohen *d* effect size was also calculated. Preplanned sensitivity analyses for this outcome included (1) PP sensitivity analysis as described above, (2) comparing 8-week scores instead of change scores, (3) multiple linear regression to adjust for potential residual confounding by baseline covariates (age, race, psychotherapy at baseline, mental health medication at baseline, previous PPD diagnosis, and previous depression diagnosis), and (4) describing scores by clinical categories. Bonferroni adjustment of *P* values was performed to account for the 4 statistical tests (primary and first 3 sensitivity) run on the PHQ-8 outcome.

The exploratory outcomes for preliminary assessment of mood and efficacy (PSS, EPDS, GAD-7, and MIBS) were summarized at each time point with Cohen *d* to assess group differences. Change scores from baseline to 8 weeks were additionally compared and summarized using the mean difference with 95% CI. Exploratory outcomes of therapeutic bond were summarized at baseline and 8-weeks.

Exploratory stratified analyses were performed to describe outcomes and the measure of perceptions of stigma by strata of age, Hispanic/Latina ethnicity, racial background, marital status, sexual orientation, employment status, education, health insurance, and neighborhood atlas. To maintain a high enough sample size for meaningful grouping, within each stratification some response options were grouped or removed. While grouping removes some important between-group differences, we opted to do this to be able to share some information rather than excluding minority groups altogether. Due to variability in circumstances related to being a student, we do not report student strata for employment and education analyses.

### Missing Data

For the primary between-group comparison of treatment effect size (secondary outcome PHQ-8), we used multiple imputation to complete any missing PHQ-8 scores to minimize potential bias from missing data. Multiple imputation by chained equations was performed using the miceforest library (version 6.0.3) [50] with the default random forests predictive model. Covariates were the same as for the multiple regression analysis, with missing data in these additionally imputed as needed: study group, age, race, psychotherapy at baseline, mental health medication at baseline, previous PPD diagnosis, and previous depression diagnosis. For single-group and exploratory outcome analyses, where the analytical goals were more descriptive or preliminary, participants who did not complete a measure in full at any given time point were excluded from pertinent analyses. We report the number of participants included at each time point for each measure.

For descriptive analyses, measures were summarized by mean and SD or by median and IQR, whether normally distributed or not, respectively. Analyses were completed in Python (version 3.8).

## Results

### Study Sample

Of 296 participants who consented, 15 (5.1%) were screen failures, and 14 (4.7%; W-PPMA: n=12, waitlist: n=2) withdrew from this study, leaving 267 participants (W-PPMA: n=144, waitlist: n=123) for analyses. There were an additional 580 fraudulent accounts detected and removed; these accounts were identified through study monitoring procedures (eg, anomalous completion times and duplicate IP addresses) and were excluded before randomization and from all analyses (Figure 2). Table 1 shows participant self-reported sociodemographic characteristics overall and by treatment group. Overall, the mean participant age was 32.2 (SD 5.1; range 16.3-48.7)years; all 267 participants identified as women, 34 (12.7%) identified as Hispanic/Latina, and 183 (68.5%) identified as White. After randomization, the W-PPMA and waitlist groups were similar on most characteristics, with notable exceptions that a greater proportion of the W-PPMA group was Hispanic/Latina (23/144, 16%, vs 11/123, 8.9%) and identified with multiple races (24/144, 16.7% vs 11/123, 8.9%) whereas the waitlist group had a higher proportion of White participants (90/123, 73.2%, vs 93/144, 64.6%), participants with a college degree (46/123, 37.4%, vs 39/144, 27.1%), and participants with an ADI ranking of at least 75 (14/123, 11.4% vs 7/144, 4.9%).

**Figure 2.**
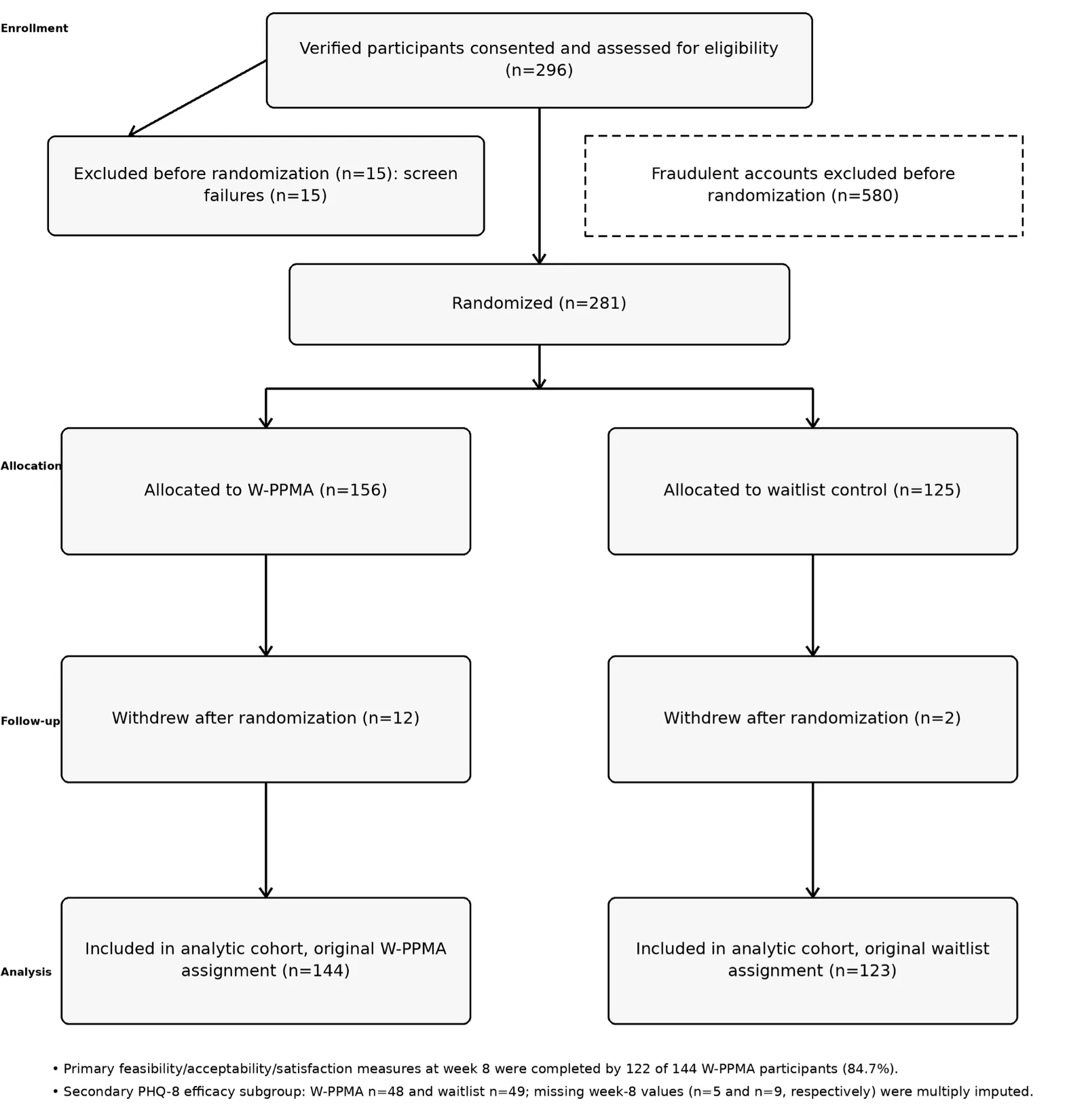
CONSORT (Consolidated Standards of Reporting Trials) flow diagram for participant recruitment.

**Table 1.** Sociodemographic characteristics of participants overall and by assigned treatment group. These self-reported survey data were collected at study baseline^a^.

	Overall (N=267)	W-PPMA^b^ (n=144)	Waitlist (n=123)
Age category (years), n (%)			
<35	190 (71.2)	103 (71.5)	87 (70.7)
≥35	76 (28.5)	40 (27.8)	36 (29.3)
Missing	1 (0.4)	1 (0.7)	0 (0.0)
Gender, n (%)			
Woman	267 (100.0)	144 (100.0)	123 (100.0)
Sex assigned at birth, n (%)			
Female	266 (99.6)	143 (99.3)	123 (100.0)
Missing	1 (0.4)	1 (0.7)	0 (0.0)
Hispanic/Latina, n (%)			
Yes	34 (12.7)	23 (16.0)	11 (8.9)
No	231 (86.5)	120 (83.3)	111 (90.2)
Missing	2 (0.7)	1 (0.7)	1 (0.8)
Racial background, n (%)			
Asian	8 (3.0)	5 (3.5)	3 (2.4)
Black/African American/African	23 (8.6)	11 (7.6)	12 (9.8)
Hispanic/Latina/Spanish^c^	15 (5.6)	11 (7.6)	4 (3.3)
White	183 (68.5)	93 (64.6)	90 (73.2)
Multiple	35 (13.1)	24 (16.7)	11 (8.9)
Missing	3 (1.1)	0 (0.0)	3 (2.4)
Marital status, n (%)			
Married/partnered/cohabitating	240 (89.9)	127 (88.2)	113 (91.9)
Divorced/separated	4 (1.5)	3 (2.1)	1 (0.8)
Single	22 (8.2)	13 (9.0)	9 (7.3)
Missing	1 (0.4)	1 (0.7)	0 (0.0)
Education, n (%)			
Graduate or postgraduate degree	107 (40.1)	60 (41.7)	47 (38.2)
College degree	85 (31.8)	39 (27.1)	46 (37.4)
Some college or technical school	50 (18.7)	27 (18.8)	23 (18.7)
High school graduate or General Educational Development degree	18 (6.7)	12 (8.3)	6 (4.9)
Some high school (grades 9‐11)	6 (2.2)	5 (3.5)	1 (0.8)
Student	4 (1.5)	2 (1.4)	2 (1.6)
Missing	1 (0.4)	1 (0.7)	0 (0.0)
Sexual orientation, n (%)			
Straight or heterosexual	230 (86.1)	126 (87.5)	104 (84.6)
Bisexual	23 (8.6)	10 (6.9)	13 (10.6)
Lesbian or gay	3 (1.1)	2 (1.4)	1 (0.8)
Asexual	2 (0.7)	1 (0.7)	1 (0.8)
Pansexual	4 (1.5)	3 (2.1)	1 (0.8)
Demisexual	1 (0.4)	0 (0.0)	1 (0.8)
Queer	2 (0.7)	0 (0.0)	2 (1.6)
Do not know	1 (0.4)	1 (0.7)	0 (0.0)
Missing	1 (0.4)	1 (0.7)	0 (0.0)
Employment status, n (%)			
Employed, full time	161 (60.3)	83 (57.6)	78 (63.4)
Employed, part time	23 (8.6)	12 (8.3)	11 (8.9)
Primary caretaker at home, not employed outside of the home	57 (21.3)	33 (22.9)	24 (19.5)
Not employed, looking for work	10 (3.7)	6 (4.2)	4 (3.3)
Not employed, not looking for work	9 (3.4)	6 (4.2)	3 (2.4)
On disability, not able to work	2 (0.7)	1 (0.7)	1 (0.8)
Student	4 (1.5)	2 (1.4)	2 (1.6)
Missing	1 (0.4)	1 (0.7)	0 (0.0)
Health insurance, n (%)			
Private insurance	188 (70.4)	101 (70.1)	87 (70.7)
Medicaid, medical assistance, or any kind of government assistance plan for those with low incomes or a disability	56 (21.0)	28 (19.4)	28 (22.8)
TRICARE or other military health care	12 (4.5)	7 (4.9)	5 (4.1)
Veterans Affairs (VA^d^; including those who have ever used or enrolled for VA health care)	1 (0.4)	1 (0.7)	0 (0.0)
Medicare for people 65 years and older, or people with certain disabilities	2 (0.7)	2 (1.4)	0 (0.0)
Do not have health insurance	5 (1.9)	2 (1.4)	3 (2.4)
Do not know	1 (0.4)	1 (0.7)	0 (0.0)
Missing	2 (0.7)	2 (1.4)	0 (0.0)
Level of access to maternity care, n (%)			
Moderate access to care/access to maternity care	245 (91.8)	133 (92.4)	112 (91.1)
Maternity care desert/low access to care	17 (6.4)	8 (5.6)	9 (7.3)
Missing	5 (1.9)	3 (2.1)	2 (1.6)
Neighborhood atlas, n (%)			
ADI_NATRANK^e^>=75	21 (7.9)	7 (4.9)	14 (11.4)
50≤ADI_NATRANK<75	82 (30.7)	42 (29.2)	40 (32.5)
25≤ADI_NATRANK<50	95 (35.6)	55 (38.2)	40 (32.5)
ADI_NATRANK<25	66 (24.7)	38 (26.4)	28 (22.8)
Missing	3 (1.1)	2 (1.4)	1 (0.8)

a The one participant missing age value answered the screening questionnaire to confirm they were aged 16 years or older but did not provide their exact date of birth.

b W-PPMA: Woebot for Postpartum Mood and Anxiety.

c Indicates participants who self-identified their race as Hispanic/Latina in addition to their ethnicity as Hispanic/Latina.

d VA: Veterans Affairs.

e ADI_Natrank: Area Deprivation Index–National Rank; derived from the Neighborhood Atlas; higher scores indicate greater neighborhood disadvantage.

Table 2 shows psychiatric and pregnancy history. Overall, 31/267 (11.6%) participants self-reported a previous PPD diagnosis and 113/267 (42.3%) a previous depression diagnosis. At study baseline, 41/267 (15.4%) of participants reported engaging in psychotherapy and 67/267 (25.1%) taking a mental health medication. The number of past pregnancies ranged from 0 to over 5, and regarding participants’ most recent pregnancy, 38/267 (14.2%) participants had their child spend time in the neonatal intensive care unit, and 22/267 (8.2%) participants reported receiving a medical and/or genetic disorder diagnosis.

**Table 2.** Participant psychiatric and pregnancy history overall and by study group. These self-reported survey data were collected at this study’s baseline.

	Overall (N=267)	W-PPMA^a^ (n=144)	Waitlist (n=123)
Psychiatric history, n (%)			
Previous postpartum depression diagnosis			
Yes	31 (11.6)	18 (12.5)	13 (10.6)
No	234 (87.6)	125 (86.8)	109 (88.6)
Prefer not to answer	1 (0.4)	0 (0.0)	1 (0.8)
Missing	1 (0.4)	1 (0.7)	0 (0.0)
Previous depression diagnosis, n (%)			
Yes	113 (42.3)	58 (40.3)	55 (44.7)
No	152 (56.9)	84 (58.3)	68 (55.3)
Prefer not to answer	1 (0.4)	1 (0.7)	0 (0.0)
Missing	1 (0.4)	1 (0.7)	0 (0.0)
Prior and concomitant medications and therapy at baseline, n (%)			
Any treatment at connect study baseline	86 (32.2)	44 (30.6)	42 (34.1)
Psychotherapy			
Yes	41 (15.4)	24 (16.7)	17 (13.8)
No	221 (82.8)	118 (81.9)	103 (83.7)
Not sure	4 (1.5)	1 (0.7)	3 (2.4)
Missing	1 (0.4)	1 (0.7)	0 (0.0)
Mental health medication			
Yes	67 (25.1)	33 (22.9)	34 (27.6)
No	198 (74.2)	109 (75.7)	89 (72.4)
Missing	2 (0.7)	2 (1.4)	0 (0.0)
Connected to or attending activities at connect study baseline	94 (35.2)	51 (35.4)	43 (35.0)
Online support groups for moms	55 (20.6)	31 (21.5)	24 (19.5)
Mommy and me or similar gatherings	26 (9.7)	14 (9.7)	12 (9.8)
Online neighborhood groups (ie, Nextdoor)	15 (5.6)	7 (4.9)	8 (6.5)
Other activity	19 (7.1)	9 (6.2)	10 (8.1)
Missing	1 (0.4)	1 (0.7)	0 (0.0)
Follow-up visits scheduled during the next 8 weeks	251 (94.0)	130 (90.3)	121 (98.4)
OB/GYN^b^	206 (77.2)	105 (72.9)	101 (82.1)
Pediatrician	179 (67.0)	89 (61.8)	90 (73.2)
Primary care doctor	40 (15.0)	22 (15.3)	18 (14.6)
Psychiatrist, therapist, psychologist, or clinical social worker	53 (19.9)	31 (21.5)	22 (17.9)
Other provider	22 (8.2)	9 (6.2)	13 (10.6)
Missing	2 (0.7)	2 (1.4)	0 (0.0)
Pregnancy history, n (%)			
Number of previous pregnancies			
0	7 (2.6)	4 (2.8)	3 (2.4)
1	116 (43.4)	66 (45.8)	50 (40.7)
2	70 (26.2)	32 (22.2)	38 (30.9)
3	38 (14.2)	21 (14.6)	17 (13.8)
4	17 (6.4)	8 (5.6)	9 (7.3)
5+	17 (6.4)	11 (7.6)	6 (4.9)
Missing	2 (0.7)	2 (1.4)	0 (0.0)
Number of term births			
0	5 (1.9)	3 (2.1)	2 (1.6)
1	142 (53.2)	74 (51.4)	68 (55.3)
2	72 (27.0)	39 (27.1)	33 (26.8)
3	25 (9.4)	14 (9.7)	11 (8.9)
4	9 (3.4)	5 (3.5)	4 (3.3)
5+	5 (1.9)	3 (2.1)	2 (1.6)
Not applicable	7 (2.6)	4 (2.8)	3 (2.4)
Missing	2 (0.7)	2 (1.4)	0 (0.0)
Number of premature births			
0	224 (83.9)	120 (83.3)	104 (84.6)
1	24 (9.0)	12 (8.3)	12 (9.8)
2	9 (3.4)	6 (4.2)	3 (2.4)
3	1 (0.4)	0 (0.0)	1 (0.8)
Not applicable	7 (2.6)	4 (2.8)	3 (2.4)
Missing	2 (0.7)	2 (1.4)	0 (0.0)
Number of miscarriages			
0	208 (77.9)	117 (81.2)	93 (75.6)
1	40 (15.0)	16 (11.1)	24 (19.5)
2	4 (1.5)	2 (1.4)	2 (1.6)
3	4 (1.5)	3 (2.1)	1 (0.8)
5+	2 (0.7)	2 (1.4)	0 (0.0)
Not applicable	7 (2.6)	4 (2.8)	3 (2.4)
Missing	2 (0.7)	2 (1.4)	0 (0.0)
Number of stillbirths			
0	257 (96.2)	137 (95.1)	120 (97.6)
1	1 (0.4)	1 (0.7)	0 (0.0)
Not applicable	7 (2.6)	4 (2.8)	3 (2.4)
Missing	2 (0.7)	2 (1.4)	0 (0.0)
Did your most recently born child spend time in the NICU^c^?			
Yes	38 (14.2)	20 (13.9)	18 (14.6)
No	222 (83.1)	119 (82.6)	103 (83.7)
Prefer not to answer	3 (1.1)	1 (0.7)	2 (1.6)
Missing	4 (1.5)	4 (2.8)	0 (0.0)
Has your most recently born child been diagnosed with any of the following?			
No	241 (90.3)	132 (91.7)	109 (88.6)
Medical condition (eg, loss, heart condition, other birth defect)	8 (3.0)	5 (3.5)	3 (2.4)
Genetic disorder (eg, Down syndrome)	1 (0.4)	0 (0.0)	1 (0.8)
Medical and genetic conditions	1 (0.4)	0 (0.0)	1 (0.8)
Other	12 (4.5)	3 (2.1)	9 (7.3)
Missing	4 (1.5)	4 (2.8)	0 (0.0)

a W-PPMA: Woebot for Postpartum Mood and Anxiety.

b OB/gyn: obstetrician/gynecologist.

c NICU: neonatal intensive care unit.

### Primary Outcomes: Feasibility, Acceptability, and Satisfaction

Table 3 shows app engagement metrics. Across the 8-week intervention, the median number of days that participants used W-PPMA was 9.0 (IQR 5.0-23.8) and 4.0 (IQR 2.0-7.0) active weeks. Participants sent a median of 217 (IQR 89-688) in-app messages and completed 6.0 (IQR 2.3-18.8) stories and 1.5 (IQR 1.0-3.0) tools.

**Table 3.** Woebot app engagement metrics. These are the primary outcome measures of app engagement among the W-PPMA^a^ group for the initial 8-week treatment period. As the W-PPMA user experience is tailored entirely to the user’s input, there is no a priori set number of stories and/or tools a user must complete; rather, each conversation with Woebot is adapted to the user in the moment with as-needed offering of stories and/or tools.

Engagement metric	Values, median (IQR)
Active days	9.0 (5.0-23.8)
Active weeks	4.0 (2.0-7.0)
Messages sent by user	217 (89-688)
Stories completed	6.0 (2.3-18.8)
Tools completed	1.5 (1.0-3.0)
Tools rated	1.0 (0.0-2.0)

a W-PPMA: Woebot for Postpartum Mood and Anxiety.

Of the intention-to-treat W-PPMA group, 122/144 (84.7%) completed the 8-week (EOI) measures for acceptability, feasibility, and satisfaction. The median score for URPI-F was 31.0 (IQR 28.0-34.0), for URPI-A was 30.0 (IQR 28.0-34.0), and for CSQ-8 was 26.0 (IQR 24.0-29.0). Score distributions are presented in Figure S1 in Multimedia Appendix 1. Slightly higher scores were seen in the PP sensitivity analysis that included 76/144 (52.8%) of the W-PPMA group, with median scores of 32.0 (IQR 29.0-36.0) for URPI-F, 31.5 (IQR 28.0-35.2) for URPI-A, and 27.0 (IQR 24.0-29.2) for CSQ-8.

### Secondary Outcome: Symptoms of Depression (PHQ-8)

There were 97 participants (n=48 W-PPMA, n=49 waitlist) with a baseline PHQ-8 score of at least 5, the minimal total score of the PHQ-8 indicating the presence of at least mild symptoms of depression. Multiple imputation was used to impute missing 8-week PHQ-8 scores for 14 participants (n=5 W-PPMA, n=9 waitlist). Overall mean change in PHQ-8 from baseline to EOI was −0.3 (SD 4.2), whereby W-PPMA averaged slight improvement (−0.7, SD 3.4) and the waitlist group averaged no change (0.0, SD 4.8). The between-group effect size was small (Cohen *d* −0.16) and not statistically significant after adjustment for multiple testing (*P*≥.99). The PP sensitivity analysis showed a greater separation of groups (W-PPMA: −1.4, SD 3.5, waitlist: 0.7, SD 3.9, adjusted *P*=.13) with medium effect size (Cohen *d* −0.57). Figure 3 shows the distribution of change scores for the intention-to-treat and PP subgroups.

**Figure 3.**
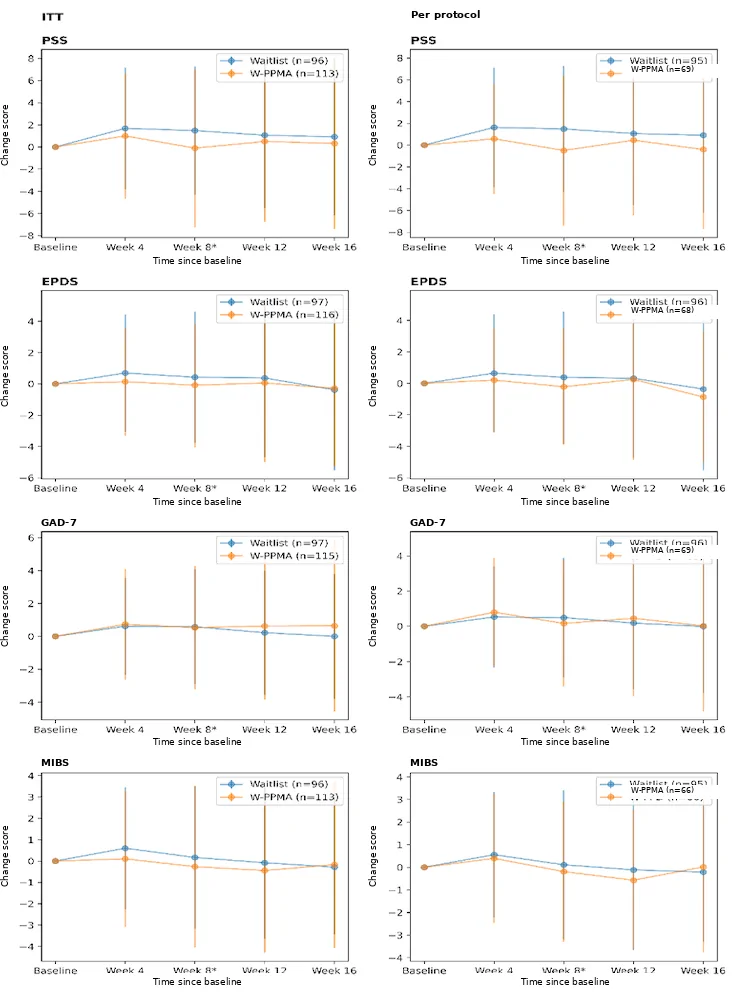
Change scores for exploratory mood and preliminary efficacy measures. Mean and SD of change scores at each time point relative to the baseline overall and by treatment group. To support interpretation in the context of the established measures, scores are presented in the original units and should not be directly compared between scales, as a 1-point change represents a different percentage change across scale ranges (PSS: 0‐40, EPDS: 0‐27, GAD-7: 0‐21, MIBS: 0‐24). The asterisk indicates the 8-week end-of-intervention point after which the waitlist group gained access to the intervention. The number in each legend is the minimum number of participants included across all time points. EPDS: Edinburgh Postnatal Depression Scale; GAD-7: Generalized Anxiety Disorder Scale; ITT: intention-to-treat; MIBS: Mother-to-Infant Bonding Scale; PSS: Perceived Stress Scale; W-PPMA: Woebot for Postpartum Mood and Anxiety.

Additional planned sensitivity analyses produced similar patterns, but neither analysis was statistically significant: the comparison of week-8 EOI scores had an adjusted *P* value of .38, and the adjusted regression analysis had an adjusted *P* value of .96. Comparison of the 8-week EOI scores alone showed lower average symptoms in the W-PPMA (mean 6.9, SD 3.4) than waitlist (8.4, SD 5.1) group (Cohen *d* −0.48, adjusted *P*=.38). Multiple regression analyses to adjust for potential residual confounding by baseline characteristics found access to W-PPMA was associated with a 1-point reduction in PHQ-8 change score as compared to the waitlist control group (95% CI −2.69 to 0.69, adjusted *P*=.96). All regression coefficients are presented in Table S1 in Multimedia Appendix 1. Examining PHQ-8 scores by clinical category showed similar distributions at baseline and descriptively lower severity in the W-PPMA group than in the waitlist group; no P value was reported for this descriptive comparison (Table S2 in Multimedia Appendix 1). Intention-to-treat results indicated that among those with elevated symptoms at baseline, 9/48 (18.8%) W-PPMA and 9/49 (18.4%) waitlist participants improved by at least 4 points. In the PP subgroup, 7 W-PPMA participants and 5 waitlist participants improved by at least 4 points.

### Exploratory Outcomes: Postnatal Anxiety (GAD-7), Depression (EPDS), Stress (PSS), MIBS, and Therapeutic Bond (WAI-SR)

Preliminary mood and efficacy measures. Table 4 displays the median (IQR) scores with Cohen *d* between-group effect size for each exploratory outcome at each time point. The PSS and EPDS showed small between-group effect sizes in favor of W-PPMA at 4-, 8-, and 12-week points. By the 16-week EOS point, differences had mostly disappeared, driven by a reduction in waitlist scores, while the W-PPMA scores stayed relatively stable after 8-weeks; the waitlist group had access to the app from 8-weeks to 16-weeks while the W-PPMA group had access for the full 16-weeks. There were very small or no differences between W-PPMA and waitlist groups across all time points for the GAD-7 and MIBS. Participant counts by measure and time point are reported in Table S3 in Multimedia Appendix 1. In the PP sensitivity analyses, differences were not statistically significant but were descriptively larger than in the primary intention-to-treat analysis, with the largest effect observed for the PSS at EOI (Cohen *d*=−0.47; Table S4 in Multimedia Appendix 1). Figure 3 shows the change scores for each measure across this study’s period. The mean difference from baseline to EOI was −1.59 (95% CI −2.4 to −0.73) for PSS, −0.51 (95% CI −1.36 to 0.34) for EPDS, −0.05 (95% CI −0.91 to 0.80) for GAD-7, and −0.43 (95% CI −1.28 to 0.42) for MIBS. For all measures except MIBS, the difference was larger in favor of W-PPMA in the PP sensitivity analyses (Table S4 legend in Multimedia Appendix 1)

Therapeutic Bond. The WAI-SR median score was 42.0 (IQR 36.0-51.0) at baseline among the 94/144 (65.3%) W-PPMA participants who completed it, and increased to 48.0 (IQR 41.0-53.0) at EOI among the 113/144 (78.5%) who completed it at the second time measure. PP sensitivity analysis scores were similar (baseline: 43.0, IQR 37.0-51.0, with n=54, week 8: 51.0, IQR 45.0-54.8, with n=70).

**Table 4.** Exploratory measures of mood and efficacy at each study time point. Median (IQR) scores with Cohen *d* measure of effect size between W-PPMA^a^ and waitlist groups for exploratory measures of mood and preliminary efficacy at each time point. Negative Cohen *d* represents lower scores in the W-PPMA group. The number of participants who completed each measure and are represented in the calculations is reported in Table S3 in Multimedia Appendix 1.

	Baseline	Week 4	Week 8	Week 12	Week 16
PSS^b^ (range 0-40)					
Overall, median (IQR)	13.0 (8.8-20.0)	15.0 (9.0-21.0)	15.0 (8.0-20.0)	14.0 (8.5-20.5)	13.5 (8.0-20.8)
W-PPMA, median (IQR)	13.0 (8.0-19.0)	15.0 (8.0-20.0)	13.0 (8.0-19.5)	13.0 (7.0-20.0)	13.0 (8.0-20.0)
Waitlist, median (IQR)	14.0 (9.0-20.0)	16.0 (10.2-22.8)	16.0 (10.0-22.0)	15.0 (10.0-22.0)	15.0 (8.0-21.0)
Cohen *d*	−0.16	−0.25	−0.33	−0.23	−0.13
EPDS^c^ (range 0-27)					
Overall, median (IQR)	5.0 (2.0-9.0)	5.0 (2.0-10.0)	5.0 (2.0-9.0)	5.0 (2.0-10.0)	4.0 (2.0-8.0)
W-PPMA, median (IQR)	5.0 (2.0-8.0)	4.0 (2.0-9.0)	4.0 (2.0-8.0)	4.0 (1.0-9.0)	4.0 (1.0-8.0)
Waitlist, median (IQR)	5.5 (2.0-10.0)	7.0 (3.0-11.0)	6.0 (2.2-10.0)	5.0 (2.0-11.0)	5.0 (2.0-8.0)
Cohen *d*	−0.16	−0.26	−0.22	−0.23	−0.10
GAD-7^d^ (range 0-21)					
Overall, median (IQR)	4.0 (2.0-7.0)	5.0 (2.0-8.0)	5.0 (2.0-8.0)	5.0 (1.0-8.0)	4.0 (1.0-7.0)
W-PPMA, median (IQR)	4.0 (2.0-7.0)	5.0 (2.0-8.0)	4.0 (1.0-7.0)	4.0 (1.0-7.8)	4.0 (1.0-7.0)
Waitlist, median (IQR)	5.0 (3.0-7.0)	5.0 (3.0-8.0)	5.0 (2.0-8.0)	5.0 (2.0-8.0)	4.0 (1.0-7.0)
Cohen *d*	−0.18	−0.11	−0.14	−0.10	0.06
MIBS^e^ (range 0-24)					
Overall, median (IQR)	3.0 (1.0-5.0)	3.0 (1.0-6.0)	3.0 (1.0-5.0)	2.0 (0.0-6.0)	2.0 (1.0-5.0)
W-PPMA, median (IQR)	3.0 (1.0-5.0)	3.0 (1.0-5.0)	2.5 (1.0-4.0)	2.0 (0.0-5.2)	2.0 (1.0-5.0)
Waitlist, median (IQR)	3.0 (1.0-5.0)	3.0 (1.0-6.0)	3.0 (1.0-6.0)	2.0 (0.0-6.0)	2.0 (0.0-5.0)
Cohen *d*	0.04	−0.14	−0.12	−0.08	0.08

a W-PPMA: Woebot for Postpartum Mood and Anxiety.

b PSS: Perceived Stress Scale.

c EPDS: Edinburgh Postnatal Depression Scale.

d GAD-7: Generalized Anxiety Disorder Scale.

e MIBS: Mother-to-Infant Bonding Scale.

### Exploratory Analyses: Stratified Analyses

#### Perceptions of Stigma

The overall baseline PBPT-Stigma Subscale score median was 12.0 (IQR 9.0-15.0), similar in W-PPMA (12.0, IQR 9.0-14.0) and waitlist (11.0, IQR 10.0-15.0) groups at baseline. Variation across strata was within 2 points. The highest point estimate was among the racial background strata of Hispanic/Latina (14.0, IQR 13.8-15.2) and those who did not have health insurance (14.0, IQR 11.5-17.5). The lowest point estimates were among those employed part time (10.0, IQR 8.0-13.5) and with military health insurance (10.0, IQR 9.0-11.5; Table S5 in Multimedia Appendix 1).

#### Usability and Acceptability Measures

The median URPI-F score was similar across strata, with the lowest median scores being 2 points below the overall median among those not employed and with ADI ranking 75 and above, and 5 points lower than the overall median among those without health insurance. Two overall strata had median scores 2 points higher, including military health care insurance and those with high school or General Educational Development (GED) education. The URPI-A had a wider range of scores across strata, with a median score 6 points above the overall median among those single, separated, or divorced and 5 points higher among ADI ranking 75 and above; and a median 6 points below the overall median among those without health insurance. The CSQ-8 had the highest median score among those with a high school or GED education and lowest among those without health insurance. All stratified usability and acceptability measure scores, including by study subgroups, are in Table S6 in Multimedia Appendix 1.

#### Preliminary Mood and Efficacy Measures

The PSS baseline scores were highest among those who reported being Black, not employed, or not having health insurance. The EOI scores were highest among those who reported being Asian, Hispanic/Latina, or not employed (Table S7 in Multimedia Appendix 1). EPDS baseline scores were highest among those who reported being Black and without health insurance, with week 8 scores additionally highest among those who reported being Black or not employed (Table S8 in Multimedia Appendix 1). GAD-7 and MIBS median scores among the entire cohort were within 2 points across all strata (Tables S9 and S10, respectively, in Multimedia Appendix 1). WAI-SR scores were highest among Black participants, and ADI ranking of at least 75 strata at baseline and among those with military health insurance or ADI ranking of at least 75 at week 8 (Table S11 in Multimedia Appendix 1).

## Discussion

### Principal Results

In this study, we assessed the feasibility, acceptability, and preliminary efficacy of the W-PPMA intervention to provide emotional support among a diverse and bilingual cohort. The results demonstrated high feasibility and acceptability among participants, as evidenced by strong scores on the URP-IR and CSQ-8. The intervention achieved notable engagement, with participants using W-PPMA for a median of 9 (IQR 5‐24) days and sending a median of 217 (IQR 89-688) messages across the 8-week intervention period.

The primary PHQ-8 outcome showed a small, nonsignificant effect in the intention-to-treat analysis (Cohen d=−0.16; P≥.99). Among exploratory outcomes at EOI, the between-group difference in PSS change favored W-PPMA and had a 95% CI that excluded 0 (mean difference −1.59, 95% CI −2.4 to −0.73), whereas the EPDS difference was not statistically significant (mean difference −0.51, 95% CI −1.36 to 0.34). Participants in the W-PPMA group had a statistically significant improvement in perceived stress relative to the waitlist control group at EOI; the between-group difference in EPDS scores was not statistically significant. PP analyses indicated moderate effect sizes, particularly for perceived stress, highlighting the potential of W-PPMA in reducing postpartum psychological distress. Therapeutic bonding with Woebot was also positively rated, suggesting the relational agent’s ability to foster a supportive user experience.

Despite challenges in achieving the full recruitment target in a timely fashion, this study successfully enrolled a diverse cohort of postpartum individuals, ensuring representation of underrepresented racial, ethnic, and socioeconomic groups. Notably, W-PPMA demonstrated strong engagement across these diverse populations, reinforcing its promise as a scalable and inclusive intervention. The findings further support W-PPMA’s potential to foster positive therapeutic engagement among groups historically underrepresented in digital health research. For example, working alliance (WAI-SR) scores were highest among Black participants and those residing in the most socioeconomically disadvantaged neighborhoods (ADI ≥75) at baseline. By week 8, Hispanic/Latina participants, those of multiple racial backgrounds, and those in areas with an ADI ranking of at least 75 demonstrated the highest WAI-SR scores, suggesting that W-PPMA effectively engaged these populations over time.

Additionally, participant satisfaction with W-PPMA was notably high among those with a high school or GED, as indicated by the CSQ-8 scores. This underscores W-PPMA’s accessibility and acceptability among individuals who may have limited access to traditional mental health services. The intervention’s ability to resonate across educational backgrounds, racial and ethnic identities, and socioeconomic strata highlights its potential as an equitable and scalable digital mental health tool.

The small effect estimates and largely nonsignificant differences should also be interpreted in the context of the study’s early enrollment closure and resulting limited statistical power. However, the overall findings suggest that digital interventions such as W-PPMA hold promise for reducing postpartum psychological distress, particularly in populations with historically limited access to mental health care.

### Comparison With Prior Work

Fitzpatrick et al [25] demonstrated that Woebot reduced anxiety and depression symptoms among young adults, and Prochaska et al [28,29] extended this work to substance use, reporting favorable usability and clinical outcomes. More recently, Suharwardy and Ramachandran [26,27] piloted Woebot in postpartum women, showing feasibility but in smaller, less diverse samples. Compared with these studies, our trial represents the largest randomized evaluation of Woebot in a postpartum cohort to date, and uniquely emphasized recruiting participants from underrepresented racial, ethnic, and socioeconomic groups. While the effect size for depressive symptoms was modest, it is consistent with early evaluations of DMHIs and underscores the importance of feasibility and acceptability outcomes as precursors to broader clinical impact. Our results extend the literature by demonstrating high engagement, satisfaction, and therapeutic alliance in a diverse postpartum population, highlighting W-PPMA’s potential to address equity gaps in perinatal mental health care. It is also notable that guided digital interventions and telemedicine-delivered psychotherapy have generally shown stronger efficacy than unguided self-help programs [51,52]. Our modest effect sizes align with this broader pattern, while the high feasibility and acceptability of W-PPMA underscore its potential as a scalable, lower-intensity option that complements rather than replaces clinician-delivered care.

### Limitations

Although our sample included greater representation of racial and ethnic minority participants than many prior digital psychotherapy and postpartum intervention trials, it nevertheless remained majority White, which may constrain generalizability. This distribution is consistent with national surveillance data showing that most postpartum samples in the United States are majority White [7] and reflects a common challenge across perinatal mental health intervention trials. Selection bias is also possible, as participants were drawn from individuals already enrolled in the PowerMom digital research platform and willing to engage in an app-based trial, which may overrepresent those who are more digitally literate or motivated to participate in research. Our study was initially designed with a larger recruitment target to support efficacy analyses; however, early closure of enrollment resulted in an underpowered sample for detecting small to moderate effect sizes. Accordingly, efficacy findings should be interpreted as exploratory, while the primary study objectives of feasibility, acceptability, and satisfaction remain adequately addressed. Despite efforts to recruit underrepresented racial and ethnic groups, challenges in reaching some populations persisted. Future studies should consider additional strategies to ensure greater representation, such as leveraging community partnerships and multilingual recruitment materials.

Second, while this study demonstrated high engagement, the intervention’s duration (8 wk) may have been insufficient to capture the full impact of W-PPMA on all outcomes, particularly for participants with more severe baseline symptoms of depression. Additionally, reliance on self-reported measures introduces the potential for response bias, and app engagement metrics may not fully capture the depth or nuance of participants’ interactions with the intervention.

Third, an unexpected challenge was fraudulent attempts to obtain participation incentives (e-gift cards). The relatively high level of participant compensation may have contributed to fraudulent enrollment attempts. The platform’s automated system was exploited by 580 fraudulent enrollments, leading to a rapid spike in participation in both the baseline study and the Woebot substudy. These accounts were detected and excluded before randomization, ensuring that the final analytic sample comprised only verified participants who met eligibility criteria. A small number of fraudulent accounts received e-gift card links before detection. We implemented anomaly detection measures, including monitoring the time taken to complete e-consent and baseline surveys and flagging irregular enrollment patterns (eg, duplicate IP addresses). These measures successfully curtailed fraudulent activity. Future studies should incorporate automated fraud detection tools from the outset to prevent disruptions and ensure data integrity.

Fourth, the lack of blinding and the use of a waitlist control group, rather than an active comparator, may have influenced participants’ responses. While ethical considerations guided this choice, future research should explore the efficacy of W-PPMA against active comparators to better contextualize its effectiveness.

Finally, attrition and missing data were challenges, particularly among participants with higher baseline depressive symptoms. Although imputation methods and sensitivity analyses were used to address this, results should be interpreted with caution.

### Future Directions

Future work should prioritize adequately powered randomized controlled trials with active comparators to rigorously assess clinical efficacy. Longer follow-up periods beyond 16 weeks will be essential to evaluate the durability of intervention effects and their potential role in relapse prevention. In parallel, implementation studies are needed to explore how W-PPMA can be integrated into postpartum care pathways, including obstetric, pediatric, and primary care settings. Real-world deployment of W-PPMA could involve integration within obstetric, pediatric, or primary care settings, where it may serve as a first-line or adjunctive support tool. As the intervention is automated, per-user delivery costs are likely to be substantially lower than clinician-delivered psychotherapy, although formal cost-effectiveness evaluations are needed. Finally, equity considerations remain central. Although our recruitment strategy successfully engaged historically underrepresented populations, additional strategies are needed to address barriers such as technology access, digital literacy, and bilingual delivery. Ensuring equitable reach and sustained engagement will be critical to maximizing the impact of DMHIs such as W-PPMA on postpartum mental health.

### Conclusions

This research demonstrates W-PPMA’s feasibility, acceptability, and potential as a scalable digital support tool for postpartum mental health in a diverse population. High user engagement and satisfaction underscore the intervention’s usability, while exploratory findings suggest benefits for depressive symptoms and perceived stress. Importantly, W-PPMA’s accessibility and cultural adaptability highlight its promise in addressing systemic barriers to postpartum mental health care, including stigma, cost, and provider shortages.

Beyond individual outcomes, these results contribute to the growing evidence base for DMHIs, extending prior work by demonstrating engagement and therapeutic alliance in a large, diverse postpartum cohort. Collectively, our findings position W-PPMA as a promising equitable solution to bridge persistent gaps in perinatal mental health support.

## Supplementary material

10.2196/74083 Multimedia Appendix 1 Outcome distributions, regression coefficients, data-completion counts, sensitivity analyses, and stratified analyses.

10.2196/74083 Checklist 1 CONSORT-eHealth Checklist.

## Abbreviations

ADI: Area Deprivation Index
CBT: cognitive behavioral therapy
CONSORT: Consolidated Standards of Reporting Trials
CSQ-8: Client Satisfaction Questionnaire–8 items
DMHI: digital mental health intervention
EOI: end of intervention
EOS: end of study
EPDS: Edinburgh Postnatal Depression Scale
GAD-7: Generalized Anxiety Disorder–7 items
GED: General Educational Development
HIPAA: Health Insurance Portability and Accountability Act
IPT: interpersonal psychotherapy
IRB: Institutional Review Board
MIBS: Mother-to-Infant Bonding Scale
NIH: National Institutes of Health
PBPT: Perceived Barriers to Psychological Treatment
PHQ-8: Patient Health Questionnaire–8 items
PP: per protocol
PPD: postpartum depression
PSS: Perceived Stress Scale–10
UBR: underrepresented in biomedical research
URP-IR: Usage Rating Profile–Intervention Revised
URPI-A: Usage Rating Profile–Intervention Revised, Acceptability Subscale
URPI-F: Usage Rating Profile–Intervention Revised, Feasibility Subscale
W-PPMA: Woebot for Postpartum Mood and Anxiety
WAI-SR: Working Alliance Inventory–Short Revised
