# Supplementary material for: Woebot for Postpartum Mood and Anxiety: Randomized Controlled Trial Evaluating Feasibility, Acceptability, and Preliminary Efficacy of a Mobile Cognitive Behavioral Therapy Intervention
Source: JMIR Pediatr Parent. 2026 Oct 9;9:e74083. doi: 10.2196/74083 (PMC13662244; doi:10.2196/74083)
Supplement: Supplementary file 1 [file pediatrics-v9-e74083-s002.pdf]

CONSORT-EHEALTH Checklist V1.6.2 Report	Manuscript Number	74083

(based on CONSORT-EHEALTH V1.6), available at [http://tinyurl.com/consort-ehealth-v1-6].

Date completed
3/17/2025 17:27:03
by
Toluwalase

TITLE

1a-i) Identify the mode of delivery in the title

1a-ii) Non-web-based components or important co-interventions in title
"Woebot for Postpartum Mood and Anxiety: A Randomized Controlled Trial Evaluating Feasibility, Acceptability, and Preliminary Efficacy of a Mobile CBT Intervention"

1a-iii) Primary condition or target group in the title

ABSTRACT

1b-i) Key features/functionalities/components of the intervention and comparator in the METHODS section of the ABSTRACT

1b-ii) Level of human involvement in the METHODS section of the ABSTRACT
"This randomized controlled trial recruited participants from the PowerMom study, a digital platform for maternal health research. Eligible individuals (≥16 years, <3 months postpartum) were randomized to Woebot for Postpartum Mood and Anxiety (W-PPMA) or a waitlist control condition. "

1b-iii) Open vs. closed, web-based (self-assessment) vs. face-to-face assessments in the METHODS section of the ABSTRACT

1b-iv) RESULTS section in abstract must contain use data

1b-v) CONCLUSIONS/DISCUSSION in abstract for negative trials

INTRODUCTION

2a-i) Problem and the type of system/solution

2a-ii) Scientific background, rationale: What is known about the (type of) system
"The postpartum period is a time of significant lifestyle, relational, and physical health changes, often accompanied by psychological distress and profound impacts on maternal mood."

METHODS

3a) CONSORT: Description of trial design (such as parallel, factorial) including allocation ratio

3b) CONSORT: Important changes to methods after trial commencement (such as eligibility criteria), with reasons
"However, few digital interventions have been rigorously studied in postpartum populations. By evaluating the feasibility, acceptability, and preliminary efficacy of W-PPMA in a diverse and bilingual cohort, this study aims to advance understanding of scalable, accessible mental health solutions. "

3b-i) Bug fixes, Downtimes, Content Changes

4a) CONSORT: Eligibility criteria for participants
"This randomized waitlist-controlled trial evaluated the Woebot for Postpartum Mood and Anxiety (W-PPMA) digital health tool. The trial consisted of three phases: Screening/Baseline, Treatment, and Follow-up. Participants were assessed at Screening/Baseline, Day 3, Week 4, Week 8 (End of Intervention [EOI]), Week 12, and Week 16 (End of Study [EOS]). All primary endpoints were evaluated at Week 8 (EOI)."

4a-i) Computer / Internet literacy

4a-ii) Open vs. closed, web-based vs. face-to-face assessments:

4a-iii) Information giving during recruitment
"recruited participants from the PowerMom study, a digital platform for maternal health research. "

4b) CONSORT: Settings and locations where the data were collected
"Third, an unexpected challenge was the fraudulent redemption of gift cards offered as participation incentives. The platform's automated system was exploited by 580 fraudulent enrollments, leading to a rapid spike in participation in both the baseline study and the Woebot Substudy. "

4b-i) Report if outcomes were (self-)assessed through online questionnaires

4b-ii) Report how institutional affiliations are displayed
"Participation spanned 16 weeks, including an 8-week intervention or waitlist control period, followed by exploratory assessments at 12- and 16-weeks. After completing baseline assessments, participants were randomized 1:1 into the W-PPMA group or the waitlist control group."

5) CONSORT: Describe the interventions for each group with sufficient details to allow replication, including how and when they were actually administered

5-i) Mention names, credential, affiliations of the developers, sponsors, and owners

5-ii) Describe the history/development process

5-iii) Revisions and updating

5-iv) Quality assurance methods

5-v) Ensure replicability by publishing the source code, and/or providing screenshots/screen-capture video, and/or providing flowcharts of the algorithms used

5-vi) Digital preservation

5-vii) Access

5-viii) Mode of delivery, features/functionalities/components of the intervention and comparator, and the theoretical framework
"English-speaking PowerMom participants received information about the Woebot sub-study through email and in-app notifications. Interested participants were directed to the -eligibility screening, which included a disclaimer about the sensitive nature of some questions."

5-ix) Describe use parameters

"Participation spanned 16 weeks, including an 8-week intervention or waitlist control period, followed by exploratory assessments at 12- and 16-weeks. After completing baseline assessments, participants were randomized 1:1 into the W-PPMA group or the waitlist control group."		
**5-x) Clarify the level of human involvement**		
**5-xi) Report any prompts/reminders used**		
**5-xii) Describe any co-interventions (incl. training/support)**		
"Assessments were conducted via the PowerMom app at Baseline, Day 3, 4-week, 8-week (EOI), 12-week, and 16-week (EOS) timepoints. Each assessment took 10–15 minutes, and participants received reminders via email and PowerMom-app push notifications."		
**6a) CONSORT: Completely defined pre-specified primary and secondary outcome measures, including how and when they were assessed**		
"Eligible participants were PowerMom enrollees aged 16 years or older, less than three months postpartum, English-literate, smartphone users, and willing to engage with the program and assessments for 16 weeks."		
**6a-i) Online questionnaires: describe if they were validated for online use and apply CHERRIES items to describe how the questionnaires were designed/deployed**		
**6a-ii) Describe whether and how "use" (including intensity of use/dosage) was defined/measured/monitored**		
**6a-iii) Describe whether, how, and when qualitative feedback from participants was obtained**		
**6b) CONSORT: Any changes to trial outcomes after the trial commenced, with reasons**		
"English-speaking PowerMom participants received information about the Woebot sub-study through email and in-app notifications. Interested participants were directed to the -eligibility screening, which included a disclaimer about the sensitive nature of some questions."		
**7a) CONSORT: How sample size was determined**		
**7a-i) Describe whether and how expected attrition was taken into account when calculating the sample size**		
**7b) CONSORT: When applicable, explanation of any interim analyses and stopping guidelines**		
"Primary Outcomes: Feasibility and Acceptability Measures<br><br>Usage Rating Profile–Intervention Revised (URP-IR): (PMID:23375174): The 6-item Feasibility and 6-item Acceptability subscales were used to measure feasibility (e.g., "The total time required to do the treatment procedures was manageable") and acceptability (e.g., "I liked the procedures used in this treatment"). Responses ranged from 1 ("strongly disagree") to 6 ("strongly agree"), with total scores ranging from 6 to 36 with higher average scores indicating greater feasibility or acceptability. The URP-IR demonstrates strong reliability, internal consistency, and discriminant validity (PMID:23375174)."		
**8a) CONSORT: Method used to generate the random allocation sequence**		
"during the enrollment period, fraudulent activity was detected. The platform's automated system was exploited by 580 fraudulent accounts created to illegitimately claim gift cards. This led to an unusual and rapid spike in enrollment across both the baseline study and the Woebot sub-study. "		
**8b) CONSORT: Type of randomisation; details of any restriction (such as blocking and block size)**		
" However, the sponsor nominated to end enrollment early (after N=296 consented) given that the a-priori goal of sample diversity (50% UBR) was successfully achieved early and also to advance time to results given the importance and relevance of the hypotheses tested herein. "		
**9) CONSORT: Mechanism used to implement the random allocation sequence (such as sequentially numbered containers), describing any steps taken to conceal the sequence until interventions were assigned**		
"After completing baseline assessments, participants were randomized 1:1 into the W-PPMA group or the waitlist control group. Neither participants nor staff were blinded to group assignments."		
**10) CONSORT: Who generated the random allocation sequence, who enrolled participants, and who assigned participants to interventions**		
"Participants randomized to W-PPMA accessed the app from randomization through 16-week (EOS) and were encouraged to use it daily, with a recommended minimum of five minutes per day during the treatment phase. Waitlist participants accessed W-PPMA starting at 8-weeks (EOI). "		
**11a) CONSORT: Blinding - If done, who was blinded after assignment to interventions (for example, participants, care providers, those assessing outcomes) and how**		
**11a-i) Specify who was blinded, and who wasn't**		
**11a-ii) Discuss e.g., whether participants knew which intervention was the "intervention of interest" and which one was the "comparator"**		
"Neither participants nor staff were blinded to group assignments."		
**11b) CONSORT: If relevant, description of the similarity of interventions**		
This was not addressed in our paper		
**12a) CONSORT: Statistical methods used to compare groups for primary and secondary outcomes**		
The full protocol is not available, but we do address data availability		
**12a-i) Imputation techniques to deal with attrition / missing values**		
**12b) CONSORT: Methods for additional analyses, such as subgroup analyses and adjusted analyses**		
this was not addressed in this paper		
RESULTS		
**13a) CONSORT: For each group, the numbers of participants who were randomly assigned, received intended treatment, and were analysed for the primary outcome**		
**13b) CONSORT: For each group, losses and exclusions after randomisation, together with reasons**		
"..assess the preliminary efficacy of W-PPMA relative to a waitlist control condition to manage symptoms of depression was analyzed among the subgroup of participants with PHQ-8 greater than or equal to 5 at baseline (minimal to several symptoms of depression). Mean eight-week change scores were calculated and compared using a two-sided independent t-test. "		
**13b-i) Attrition diagram**		
**14a) CONSORT: Dates defining the periods of recruitment and follow-up**		
"Of 296 participants that consented, n=15 (5.1%) were screen fails, and n=14 (4.7%; W-PPMA: n=12, Waitlist: n=2) withdrew from the study, leaving 267 participants (W-PPMA: n=144, Waitlist: n=123) for analyses. "		
**14a-i) Indicate if critical "secular events" fell into the study period**		
**14b) CONSORT: Why the trial ended or was stopped (early)**		
"Of 296 participants that consented, n=15 (5.1%) were screen fails, and n=14 (4.7%; W-PPMA: n=12, Waitlist: n=2) withdrew from the study, leaving 267 participants (W-PPMA: n=144, Waitlist: n=123) for analyses. "		
**15) CONSORT: A table showing baseline demographic and clinical characteristics for each group**		
"The recruitment period was from May 2021 and ended May 2024. initial goal was to recruit 225 participants in each arm of the study."		
**15-i) Report demographics associated with digital divide issues**		

**16a) CONSORT: For each group, number of participants (denominator) included in each analysis and whether the analysis was by original assigned groups**		
**16-i) Report multiple "denominators" and provide definitions**		
**16-ii) Primary analysis should be intent-to-treat**		
"Preliminary mood and efficacy measures. Table 3 displays the median (Q1, Q3) scores with Cohen's d between-group effect size for each exploratory outcome at each time point. The PSS and EPDS showed small between-group effect sizes in favor of W-PPMA at 4-, 8-, and 12-week points. "		
**17a) CONSORT: For each primary and secondary outcome, results for each group, and the estimated effect size and its precision (such as 95% confidence interval)**		
"However, the sponsor nominated to end enrollment early (after N=296 consented) given that the a-priori goal of sample diversity (50% UBR) ..."		
**17a-i) Presentation of process outcomes such as metrics of use and intensity of use**		
**17b) CONSORT: For binary outcomes, presentation of both absolute and relative effect sizes is recommended**		
Table 1 shows a comprehensive demographic and clinical characteristics		
**18) CONSORT: Results of any other analyses performed, including subgroup analyses and adjusted analyses, distinguishing pre-specified from exploratory**		
" for preliminary assessment of mood and efficacy (PSS, EPDS, GAD-7, MIBS) were summarized at each time point with Cohen's d to assess between group differences."		
**18-i) Subgroup analysis of comparing only users**		
**19) CONSORT: All important harms or unintended effects in each group**		
This is very detailed out in our results section as we go through the primary and secondary outcomes		
**19-i) Include privacy breaches, technical problems**		
**19-ii) Include qualitative feedback from participants or observations from staff/researchers**		
DISCUSSION		
**20) CONSORT: Trial limitations, addressing sources of potential bias, imprecision, multiplicity of analyses**		
**20-i) Typical limitations in ehealth trials**		
**21) CONSORT: Generalisability (external validity, applicability) of the trial findings**		
**21-i) Generalizability to other populations**		
**21-ii) Discuss if there were elements in the RCT that would be different in a routine application setting**		
**22) CONSORT: Interpretation consistent with results, balancing benefits and harms, and considering other relevant evidence**		
**22-i) Restate study questions and summarize the answers suggested by the data, starting with primary outcomes and process outcomes (use)**		
**22-ii) Highlight unanswered new questions, suggest future research**		
"In this study, we assessed the feasibility, acceptability, and preliminary efficacy of the Woebot for Postpartum Mood and Anxiety (W-PPMA) intervention to provide emotional support among a diverse and bilingual cohort. Results demonstrated high feasibility and acceptability among participants, as evidenced by strong scores on the Usage Rating Profile–Intervention Revised (URP-IR) and Client Satisfaction Questionnaire (CSQ-8). The intervention achieved notable engagement, with participants using W-PPMA for a median of nine days and sending an average of 217 messages across the 8-week intervention period."		
Other information		
**23) CONSORT: Registration number and name of trial registry**		
These analysis were detailed in our exploratory outcome analysis		
**24) CONSORT: Where the full trial protocol can be accessed, if available**		
This study had no harms to report		
**25) CONSORT: Sources of funding and other support (such as supply of drugs), role of funders**		
Yes-Trial Registration<br>Clinicaltrials.gov NCT05662605		
**X26-i) Comment on ethics committee approval**		
**x26-ii) Outline informed consent procedures**		
"This study was approved by the Institutional Review Board (IRB) of the Scripps Research Translational Institute."		
**X26-iii) Safety and security procedures**		
**X27-i) State the relation of the study team towards the system being evaluated**		
