# Supplementary material for: Woebot for Postpartum Mood and Anxiety: Randomized Controlled Trial Evaluating Feasibility, Acceptability, and Preliminary Efficacy of a Mobile Cognitive Behavioral Therapy Intervention
Source: JMIR Pediatr Parent. 2026 Oct 9;9:e74083. doi: 10.2196/74083 (PMC13662244; doi:10.2196/74083)
Supplement: Supplementary file 2 [file pediatrics-v9-e74083-s001.docx]

**Supplementary Material**

# Figures

**Figure S1.** Distributions of the Primary Outcome Measures of Usability and Acceptability. Left: W-PPMA=WoeBot for Postpartum Mood and Anxiety ITT assigned group who completed the measures. Right: Per Protocol subgroup sensitivity analysis.

# Tables

| **Variable** | **Coefficient** | **SE** | **P-value** | **95% CI Lower** | **95% CI Upper** |
| --- | --- | --- | --- | --- | --- |
| Intercept | 0.969 | 2.851 | 0.735 | -4.7 | 6.638 |
| W-PPMA | -1.004 | 0.849 | 0.241 | -2.692 | 0.685 |
| Race[Black/African American/African] | 0.407 | 1.259 | 0.748 | -2.098 | 2.911 |
| Race[Hispanic/Latina/Spanish] | -3.808 | 1.832 | 0.041 | -7.451 | -0.165 |
| Race[Asian] | 0.091 | 4.115 | 0.982 | -8.093 | 8.275 |
| Race[Multiple] | 0.666 | 1.255 | 0.597 | -1.83 | 3.162 |
| Psychotherapy[NotSure] | 4.75 | 2.603 | 0.072 | -0.427 | 9.926 |
| Psychotherapy[Yes] | 0.416 | 1.061 | 0.696 | -1.694 | 2.525 |
| Mental Health Medication[Yes] | -0.266 | 1.051 | 0.801 | -2.357 | 1.824 |
| Previous PPD[Prefer not to answer] | -3.589 | 4.131 | 0.387 | -11.804 | 4.626 |
| Previous PPD[Yes] | -1.11 | 1.25 | 0.377 | -3.596 | 1.376 |
| Previous Depression Diagnosis[Yes] | 2.432 | 0.958 | 0.013 | 0.528 | 4.336 |
| Age | -0.064 | 0.079 | 0.419 | -0.222 | 0.093 |

**Table S1.** Regression Coefficients from Sensitivity Analysis on PHQ-8 Baseline to 8-week Change Scores W-PPMA vs Waitlist. Multiple imputation by chained equations was used to complete meeting data for the n=97 participants included in the analysis (n=14 PHQ-8 8-week scores, n=1 age, n=2 Race values imputed). *Legend:* SE = Standard Error, PPD = Postpartum Depression.

|  | **Overall** | **W-PPMA** | **Waitlist** |
| --- | --- | --- | --- |
| **Number of participants** | 97 | 48 | 49 |
| **Baseline** |  |  |  |
| None | 0 (0.0%) | 0 (0.0%) | 0 (0.0%) |
| Minimal | 74 (76.3%) | 38 (79.2%) | 36 (73.5%) |
| Moderate | 15 (15.5%) | 7 (14.6%) | 8 (16.3%) |
| Moderately severe | 8 (8.2%) | 3 (6.2%) | 5 (10.2%) |
| Severe | 0 (0.0%) | 0 (0.0%) | 0 (0.0%) |
| **Week 8** |  |  |  |
| None | 24 (24.7%) | 13 (27.1%) | 11 (22.4%) |
| Minimal | 46 (47.4%) | 25 (52.1%) | 21 (42.9%) |
| Moderate | 20 (20.6%) | 8 (16.7%) | 12 (24.5%) |
| Moderately severe | 4 (4.1%) | 2 (4.2%) | 2 (4.1%) |
| Severe | 3 (3.1%) | 0 (0.0%) | 3 (6.1%) |

**Table S2.** PHQ-8 Sensitivity Analysis Clinical Categories. This sensitivity analysis categorizes and describes the PHQ-8 scores according to severity of depressive symptoms overall and by group: 0–4 (minimal/no depression), 5–9 (minimal depression), 10–14 (moderate depression), 15–19 (moderately severe depression), 20–24 (severe depression).

|  | **Baseline**  **n (%)** | **Week 4**  **n (%)** | **Week 8**  **n (%)** | **Week 12**  **n (%)** | **Week 16**  **n (%)** |
| --- | --- | --- | --- | --- | --- |
| **Perceived Stress Scale (PSS)** | | | | | |
| **Intention to Treat Analysis** | |  |  |  |  |
| Overall | 260 (97.4%) | 233 (87.3%) | 225 (84.3%) | 219 (82.0%) | 214 (80.1%) |
| W-PPD | 139 (96.5%) | 123 (85.4%) | 123 (85.4%) | 120 (83.3%) | 117 (81.2%) |
| Waitlist | 121 (98.4%) | 110 (89.4%) | 102 (82.9%) | 99 (80.5%) | 97 (78.9%) |
| **Per Protocol Sensitivity Analysis** | | |  |  |  |
| Overall | 201 (75.3%) | 185 (69.3%) | 178 (66.7%) | 219 (82.0%) | 213 (79.8%) |
| W-PPD | 81 (56.2%) | 75 (52.1%) | 76 (52.8%) | 120 (83.3%) | 116 (80.6%) |
| Waitlist | 120 (97.6%) | 110 (89.4%) | 102 (82.9%) | 99 (80.5%) | 97 (78.9%) |
| **The Edinburgh Postnatal Depression Scale (EPDS)** | | |  |  |  |
| **Intention to Treat Analysis** | |  |  |  |  |
| Overall | 263 (98.5%) | 232 (86.9%) | 223 (83.5%) | 221 (82.8%) | 216 (80.9%) |
| W-PPD | 141 (97.9%) | 123 (85.4%) | 121 (84.0%) | 122 (84.7%) | 118 (81.9%) |
| Waitlist | 122 (99.2%) | 109 (88.6%) | 102 (82.9%) | 99 (80.5%) | 98 (79.7%) |
| **Per Protocol Sensitivity Analysis** | | |  |  |  |
| Overall | 201 (75.3%) | 184 (68.9%) | 176 (65.9%) | 221 (82.8%) | 215 (80.5%) |
| W-PPD | 81 (56.2%) | 75 (52.1%) | 74 (51.4%) | 122 (84.7%) | 117 (81.2%) |
| Waitlist | 120 (97.6%) | 109 (88.6%) | 102 (82.9%) | 99 (80.5%) | 98 (79.7%) |
| **The Generalized Anxiety Disorder Scale (GAD-7)** | | |  |  |  |
| **Intention to Treat Analysis** | |  |  |  |  |
| Overall | 263 (98.5%) | 232 (86.9%) | 222 (83.1%) | 221 (82.8%) | 215 (80.5%) |
| W-PPD | 140 (97.2%) | 122 (84.7%) | 121 (84.0%) | 122 (84.7%) | 118 (81.9%) |
| Waitlist | 123 (100.0%) | 110 (89.4%) | 101 (82.1%) | 99 (80.5%) | 97 (78.9%) |
| **Per Protocol Sensitivity Analysis** | | |  |  |  |
| Overall | 202 (75.7%) | 185 (69.3%) | 175 (65.5%) | 221 (82.8%) | 214 (80.1%) |
| W-PPD | 81 (56.2%) | 75 (52.1%) | 74 (51.4%) | 122 (84.7%) | 117 (81.2%) |
| Waitlist | 121 (98.4%) | 110 (89.4%) | 101 (82.1%) | 99 (80.5%) | 97 (78.9%) |
| **The Mother-to-Infant Bonding Scale (MIB)** | | |  |  |  |
| **Intention to Treat Analysis** | |  |  |  |  |
| Overall | 259 (97.0%) | 230 (86.1%) | 224 (83.9%) | 219 (82.0%) | 215 (80.5%) |
| W-PPD | 138 (95.8%) | 121 (84.0%) | 122 (84.7%) | 120 (83.3%) | 118 (81.9%) |
| Waitlist | 121 (98.4%) | 109 (88.6%) | 102 (82.9%) | 99 (80.5%) | 97 (78.9%) |
| **Per Protocol Sensitivity Analysis** | | |  |  |  |
| Overall | 198 (74.2%) | 183 (68.5%) | 178 (66.7%) | 219 (82.0%) | 214 (80.1%) |
| W-PPD | 79 (54.9%) | 74 (51.4%) | 76 (52.8%) | 120 (83.3%) | 117 (81.2%) |
| Waitlist | 119 (96.7%) | 109 (88.6%) | 102 (82.9%) | 99 (80.5%) | 97 (78.9%) |

**Table S3.** Exploratory Mood and Efficacy Measure Completeness. This table described the number of participants (n) and percentage of relevant group that completed each of the EPDS, PSS, GAD-7, and MIB at each time point.

|  | **Baseline** | **Week 4** | **Week 8** | **Week 12** | **Week 16** |
| --- | --- | --- | --- | --- | --- |
| **Perceived Stress Scale (PSS) [Range 0,40]** | | | | | |
| **Overall** | 13.0 (9.0, 19.0) | 15.0 (8.0, 21.0) | 14.0 (8.0, 20.0) | 14.0 (8.5, 20.5) | 14.0 (8.0, 21.0) |
| **W-PPD** | 12.0 (8.0, 17.0) | 13.0 (7.5, 18.0) | 12.0 (5.8, 17.2) | 13.0 (7.0, 20.0) | 13.0 (8.0, 20.2) |
| **Waitlist** | 14.0 (9.0, 20.0) | 16.0 (10.2, 22.8) | 16.0 (10.0, 22.0) | 15.0 (10.0, 22.0) | 15.0 (8.0, 21.0) |
| **Cohen's d** | -0.26 | -0.36 | -0.47 | -0.23 | -0.12 |
| **The Edinburgh Postnatal Depression Scale (EPDS) [Range 0, 27]** | | | | | |
| **Overall** | 5.0 (2.0, 9.0) | 5.0 (2.0, 9.2) | 5.0 (2.0, 9.0) | 5.0 (2.0, 10.0) | 4.0 (2.0, 8.0) |
| **W-PPD** | 4.0 (2.0, 7.0) | 4.0 (2.0, 7.0) | 3.0 (2.0, 8.0) | 4.0 (1.0, 9.0) | 4.0 (1.0, 8.0) |
| **Waitlist** | 6.0 (2.0, 10.0) | 7.0 (3.0, 11.0) | 6.0 (2.2, 10.0) | 5.0 (2.0, 11.0) | 5.0 (2.0, 8.0) |
| **Cohen's d** | -0.26 | -0.33 | -0.32 | -0.23 | -0.10 |
| **The Generalized Anxiety Disorder Scale (GAD-7) [Range 0,21]** | | | | | |
| **Overall** | 4.0 (2.0, 7.0) | 5.0 (2.0, 8.0) | 5.0 (2.0, 8.0) | 5.0 (1.0, 8.0) | 4.0 (1.0, 7.0) |
| **W-PPD** | 4.0 (2.0, 6.0) | 4.0 (2.0, 8.0) | 3.5 (1.0, 6.0) | 4.0 (1.0, 7.8) | 4.0 (1.0, 7.0) |
| **Waitlist** | 5.0 (3.0, 7.0) | 5.0 (3.0, 8.0) | 5.0 (2.0, 8.0) | 5.0 (2.0, 8.0) | 4.0 (1.0, 7.0) |
| **Cohen's d** | -0.25 | -0.14 | -0.24 | -0.10 | 0.06 |
| **The Mother-to-Infant Bonding Scale (MIBS) [Range 0, 24]** | | | | | |
| **Overall** | 3.0 (1.0, 5.0) | 3.0 (1.0, 6.0) | 3.0 (1.0, 5.0) | 2.0 (0.0, 6.0) | 2.0 (1.0, 5.0) |
| **W-PPD** | 3.0 (1.0, 5.0) | 3.0 (1.2, 5.0) | 2.5 (1.0, 4.0) | 2.0 (0.0, 5.2) | 2.0 (1.0, 5.0) |
| **Waitlist** | 3.0 (1.0, 5.0) | 3.0 (1.0, 6.0) | 3.0 (1.0, 6.0) | 2.0 (0.0, 6.0) | 2.0 (0.0, 5.0) |
| **Cohen's d** | -0.11 | -0.16 | -0.25 | -0.08 | 0.09 |

**Table S4.** Per protocol sensitivity analysis Median (Quartile 1, Quartile 3) scores at each time point with Cohen's D measure of effect size between W-PPD and Waitlist Groups for exploratory measures of mood and preliminary efficacy. The mean difference (95% confidence interval) for change from baseline to week 8 was -1.98 (95% CI: -2.92, -1.04) for PSS, -0.60 (95% CI: -1.54, 0.34) for EPDS, -0.33 (95% CI: -1.27, 0.62) for GAD-7 , and -0.3 (95% CI: -1.24, 0.64) for MIB.

| **Variable** | **Overall** | **W-PPMA** | **Waitlist** |
| --- | --- | --- | --- |
| **Overall** | 12.0 (9.0, 15.0) | 12.0 (9.0, 14.0) | 11.0 (10.0, 15.0) |
| Missing (n) | 46 | 28 | 18 |
| **Age category** |  |  |  |
| Missing (n) | 1 | 1 | 0 |
| <35 | 12.0 (9.2, 15.0) | 12.0 (9.0, 14.2) | 12.0 (10.0, 15.0) |
| >=35 | 11.0 (9.0, 13.0) | 11.0 (9.0, 13.5) | 11.0 (9.0, 13.0) |
| **Hispanic or Latina** |  |  |  |
| Missing (n) | 2 | 1 | 1 |
| No | 11.0 (9.0, 14.8) | 11.0 (9.0, 14.0) | 11.0 (9.0, 15.0) |
| Yes | 12.0 (11.0, 14.0) | 13.0 (11.0, 14.5) | 12.0 (11.0, 13.0) |
| **Racial background** |  |  |  |
| Missing (n) | 3 | 0 | 3 |
| Asian | 13.0 (11.0, 15.0) | 12.5 (11.5, 14.0) | 13.0 (11.5, 15.0) |
| Black/African American/African | 11.5 (8.0, 14.2) | 12.0 (10.0, 14.5) | 11.0 (8.0, 13.8) |
| Hispanic/Latina/Spanish | 14.0 (13.8, 15.2) | 14.0 (13.2, 15.0) | 16.5 (15.2, 17.8) |
| Multiple | 11.0 (10.0, 13.0) | 11.0 (9.5, 13.0) | 12.0 (11.0, 12.0) |
| White | 11.0 (9.0, 14.2) | 11.0 (8.0, 14.0) | 11.0 (9.0, 15.0) |
| **Marital status** |  |  |  |
| Missing (n) | 1 | 1 | 0 |
| Married/Partnered/Cohabitating | 12.0 (9.0, 14.5) | 12.0 (9.0, 14.0) | 11.0 (10.0, 15.0) |
| Single/Separated/Divorced | 12.0 (9.0, 14.8) | 12.0 (8.2, 14.5) | 11.5 (9.8, 14.2) |
| **Sexual Orientation** |  |  |  |
| Missing (n) | 1 | 1 | 0 |
| Sexual minority | 11.0 (8.0, 14.0) | 9.5 (7.0, 13.0) | 12.0 (10.0, 14.5) |
| Straight or heterosexual | 12.0 (9.0, 15.0) | 12.0 (9.0, 14.0) | 11.0 (9.2, 15.0) |
| **Employment status** |  |  |  |
| Missing (n) | 7 | 4 | 3 |
| Employed, full time | 11.0 (9.0, 14.0) | 11.0 (9.0, 13.0) | 11.0 (9.0, 15.0) |
| Employed, part time | 10.0 (8.0, 13.5) | 9.0 (8.0, 13.0) | 10.5 (10.0, 15.0) |
| Not employed | 13.0 (11.0, 16.0) | 13.0 (11.5, 16.5) | 12.0 (10.2, 13.8) |
| Primary caretaker at home, not employed outside of the home | 12.0 (9.0, 15.0) | 12.0 (9.0, 15.0) | 12.0 (9.0, 15.5) |
| **Education** |  |  |  |
| Missing (n) | 1 | 1 | 0 |
| College degree | 12.0 (10.0, 14.8) | 12.0 (9.5, 14.5) | 12.0 (10.0, 14.5) |
| Graduate or postgraduate degree | 11.0 (9.0, 13.0) | 11.0 (8.5, 13.0) | 10.0 (9.0, 12.0) |
| High school or GED | 11.0 (8.0, 16.0) | 11.5 (7.0, 15.2) | 10.0 (9.0, 17.0) |
| Some college or technical school | 12.5 (10.0, 16.2) | 12.0 (9.2, 14.0) | 14.0 (11.0, 18.8) |
| **Health insurance** |  |  |  |
| Missing (n) | 3 | 3 | 0 |
| Do not have health insurance | 14.0 (11.5, 17.5) | 9.0 (9.0, 9.0) | 17.5 (15.8, 19.2) |
| Medicaid, Medicare, or other government assistance plan | 12.0 (10.0, 15.5) | 12.0 (10.0, 14.5) | 13.0 (9.8, 18.2) |
| Private insurance | 11.5 (9.0, 14.0) | 12.0 (9.0, 14.0) | 11.0 (10.0, 13.5) |
| TRICARE, VA, or other military health care | 10.0 (9.0, 11.5) | 10.0 (9.5, 11.0) | 10.5 (8.5, 12.8) |
| **Neighborhood atlas** |  |  |  |
| Missing (n) | 3 | 2 | 1 |
| 25 <= ADI_NATRANK < 50 | 11.0 (9.0, 14.8) | 12.0 (9.0, 15.0) | 10.0 (9.0, 13.0) |
| 50 <= ADI_NATRANK < 75 | 12.0 (10.0, 14.2) | 12.0 (10.0, 14.0) | 11.0 (10.0, 14.5) |
| ADI_NATRANK < 25 | 12.0 (9.0, 14.0) | 9.5 (8.0, 13.0) | 13.0 (11.0, 17.0) |
| ADI_NATRANK >= 75 | 12.5 (10.2, 16.8) | 12.0 (12.0, 15.0) | 13.0 (9.5, 18.5) |

**Table S5.** PBC-Stigma Stratified Scores at Day 3. Numbers represent median (quartile 1, quartile 3) scores unless otherwise specified whereby n = Number of Participants. **Legend:** W-PPMA: WoeBot for Postpartum Mood and Anxiety, n = Number of Participants, GED = General Education Degree, VA = Veterans Affairs, ADI_Natrank = Area Deprivation Index - National Rank.

| **Variable** | **URPI-F** | **URPI-A** | **CSQ-8** |
| --- | --- | --- | --- |
| **Overall** | 31.00 (28.00, 34.00) | 30.00 (28.00, 34.00) | 26.00 (24.00, 29.00) |
| Missing (n) | 22 | 22 | 22 |
| **Age category** |  |  |  |
| Missing | 1 | 1 | 1 |
| <35 | 32.00 (28.00, 35.00) | 30.00 (28.00, 34.00) | 27.00 (24.00, 30.00) |
| >=35 | 30.00 (27.50, 33.00) | 30.00 (29.00, 34.50) | 26.00 (23.50, 29.00) |
| **Hispanic or Latina** |  |  |  |
| Missing (n) | 1 | 1 | 1 |
| No | 31.00 (28.00, 34.00) | 30.00 (28.00, 34.00) | 26.00 (24.00, 29.00) |
| Yes | 31.00 (28.00, 34.00) | 30.00 (30.00, 32.00) | 28.00 (25.00, 30.00) |
| **Racial background** |  |  |  |
| Missing (n) | 0 | 0 | 0 |
| Asian | 30.00 (27.00, 32.00) | 30.00 (28.00, 30.00) | 25.00 (24.00, 25.00) |
| Black/African American/African | 31.50 (30.00, 33.00) | 32.00 (24.75, 36.00) | 27.00 (24.50, 31.25) |
| Hispanic/Latina/Spanish | 31.00 (30.00, 33.50) | 30.50 (30.00, 32.00) | 28.00 (24.75, 29.75) |
| Multiple | 32.00 (29.00, 36.00) | 31.00 (28.25, 33.50) | 27.50 (24.50, 30.00) |
| White | 31.00 (28.00, 34.00) | 30.00 (28.00, 34.50) | 26.00 (24.00, 29.00) |
| **Marital status** |  |  |  |
| Missing (n) | 1 | 1 | 1 |
| Married/Partnered/Cohabitate | 31.00 (28.00, 34.00) | 30.00 (28.00, 32.25) | 26.00 (24.00, 29.00) |
| Single/Separated/Divorced | 32.00 (28.25, 34.75) | 36.00 (32.00, 36.00) | 28.50 (27.00, 32.00) |
| **Sexual Orientation** |  |  |  |
| Missing (n) | 1 | 1 | 1 |
| Sexual minority | 31.00 (28.50, 32.50) | 31.00 (29.00, 33.50) | 27.00 (23.50, 29.00) |
| Straight or heterosexual | 31.00 (28.00, 35.00) | 30.00 (28.00, 34.00) | 26.00 (24.00, 29.00) |
| **Employment status** |  |  |  |
| Missing (n) | 4 | 4 | 4 |
| Employed, full time | 31.00 (28.00, 35.00) | 30.00 (27.00, 32.00) | 25.00 (23.00, 28.00) |
| Employed, part time | 32.00 (26.25, 34.00) | 31.00 (25.25, 36.00) | 25.50 (21.75, 31.25) |
| Not employed | 29.00 (28.50, 31.00) | 32.00 (30.50, 34.50) | 27.00 (25.00, 28.50) |
| Primary caretaker at home, not employed outside of the home | 32.00 (30.00, 34.75) | 30.50 (29.25, 36.00) | 28.00 (25.50, 30.75) |
| **Education** |  |  |  |
| Missing (n) | 1 | 1 | 1 |
| College degree | 30.00 (27.50, 34.00) | 30.00 (28.50, 32.00) | 26.00 (24.00, 28.50) |
| Graduate or postgraduate degree | 31.00 (27.75, 34.25) | 30.00 (27.00, 32.00) | 25.00 (23.00, 27.00) |
| High school or GED | 33.00 (30.00, 36.00) | 34.00 (30.00, 36.00) | 30.00 (28.00, 32.00) |
| Some college or technical school | 31.00 (29.00, 33.00) | 31.50 (27.75, 35.00) | 27.50 (24.25, 30.00) |
| **Health insurance** |  |  |  |
| Missing (n) | 3 | 3 | 3 |
| Do not have health insurance | 26.00 (25.50, 26.50) | 24.00 (21.00, 27.00) | 22.00 (19.00, 25.00) |
| Medicaid, Medicare, or other government assistance plan | 31.00 (29.00, 34.50) | 34.00 (30.00, 36.00) | 28.00 (24.50, 30.50) |
| Private insurance | 31.00 (28.00, 34.00) | 30.00 (27.00, 32.00) | 26.00 (24.00, 28.00) |
| TRICARE, VA, or other military health care | 33.00 (29.75, 35.50) | 32.00 (30.50, 35.00) | 28.00 (27.00, 31.25) |
| **Neighborhood atlas** |  |  |  |
| Missing (n) | 2 | 2 | 2 |
| 25 <= ADI_NATRANK < 50 | 31.50 (28.00, 33.75) | 30.00 (29.00, 36.00) | 25.50 (23.00, 30.00) |
| 50 <= ADI_NATRANK < 75 | 31.00 (30.00, 36.00) | 31.00 (30.00, 33.50) | 27.00 (24.00, 29.00) |
| ADI_NATRANK < 25 | 30.50 (26.25, 34.00) | 29.50 (27.00, 30.75) | 25.00 (24.00, 27.00) |
| ADI_NATRANK >= 75 | 29.00 (28.00, 36.00) | 35.00 (28.00, 36.00) | 29.00 (28.50, 32.00) |

**Table S6.** Usability and Acceptability (URPI-F, URPI-A, CSQ-8) Stratified Scores. Numbers represent median (quartile 1, quartile 3) scores unless otherwise specified whereby n = Number of Participants. Legend: W-PPMA: WoeBot for Postpartum Mood and Anxiety, n = Number of Participants, GED = General Education Degree, VA = Veterans Affairs, ADI_Natrank = Area Deprivation Index - National Rank.

|  | **Baseline** | | | **Week 8** | | |
| --- | --- | --- | --- | --- | --- | --- |
| **Variable** | **Overall** | **W-PPMA** | **Waitlist** | **Overall** | **W-PPMA** | **Waitlist** |
| **Overall** | 13.0  (8.8, 20.0) | 13.0  (8.0, 19.0) | 14.0  (9.0, 20.0) | 15.0  (8.0, 20.0) | 13.0  (8.0, 19.5) | 16.0  (10.0, 22.0) |
| Missing (n) | 7 | 5 | 2 | 42 | 21 | 21 |
| **Age category** |  |  |  |  |  |  |
| Missing | 1 | 1 | 0 | 1 | 1 | 0 |
| <35 | 14.0  (9.0, 20.0) | 13.0  (8.0, 19.0) | 15.0  (10.0, 21.0) | 15.0  (8.0, 21.2) | 13.0  (8.0, 19.8) | 16.0  (10.2, 22.0) |
| >=35 | 13.0  (8.0, 19.8) | 12.0  (8.0, 19.8) | 13.0  (7.8, 18.5) | 14.5  (8.8, 18.2) | 12.0  (5.0, 17.2) | 15.0  (10.0, 19.0) |
| **Hispanic or Latina** |  |  |  |  |  |  |
| Missing (n) | 2 | 1 | 1 | 2 | 1 | 1 |
| No | 13.0  (8.2, 19.8) | 13.0  (8.0, 19.0) | 14.0  (9.0, 20.0) | 14.5  (8.0, 20.0) | 12.0  (7.0, 19.0) | 16.0  (10.0, 22.0) |
| Yes | 13.0  (9.0, 20.0) | 13.0  (7.2, 19.2) | 16.0  (12.0, 20.0) | 15.0  (9.8, 20.0) | 14.5  (9.2, 21.5) | 15.0  (11.8, 17.5) |
| **Racial background** |  |  |  |  |  |  |
| Missing (n) | 3 | 0 | 3 | 3 | 0 | 3 |
| Asian | 13.0  (9.2, 16.2) | 12.0  (12.0, 14.0) | 14.0  (7.5, 24.0) | 18.0  (15.0, 21.0) | 20.0  (16.0, 24.0) | 17.0  (12.0, 18.0) |
| Black/African American/African | 19.0  (13.5, 24.5) | 16.0  (14.0, 23.5) | 19.5  (12.5, 30.0) | 17.0  (11.5, 23.0) | 13.5  (9.0, 16.5) | 23.0  (17.2, 29.0) |
| Hispanic/Latina/Spanish | 16.0  (6.5, 21.0) | 13.0  (6.5, 20.0) | 20.0  (13.8, 23.8) | 18.0  (8.8, 22.0) | 20.0  (9.5, 22.5) | 14.0  (10.0, 17.0) |
| Multiple | 13.0  (8.8, 18.5) | 12.0  (8.0, 17.0) | 16.0  (10.5, 20.0) | 12.0  (8.0, 18.0) | 10.0  (5.8, 16.5) | 15.0  (10.0, 21.0) |
| White | 13.0  (8.0, 19.0) | 13.0  (8.0, 19.0) | 13.0  (9.0, 18.2) | 14.0  (8.0, 19.2) | 12.0  (6.0, 18.0) | 16.0  (10.0, 21.0) |
| **Marital status** |  |  |  |  |  |  |
| Missing (n) | 1 | 1 | 0 | 1 | 1 | 0 |
| Married/Partnered/Cohabitating | 13.0  (8.0, 19.0) | 13.0  (8.0, 19.0) | 13.0  (9.0, 19.5) | 14.0  (8.2, 20.0) | 12.0  (8.0, 20.0) | 16.0  (10.0, 20.0) |
| Single/Separated/Divorced | 15.5  (10.0, 22.0) | 14.5  (9.8, 21.2) | 17.5  (15.0, 24.2) | 17.0  (9.0, 23.0) | 16.0  (5.8, 18.0) | 23.0  (17.0, 31.0) |
| **Sexual Orientation** |  |  |  |  |  |  |
| Missing (n) | 1 | 1 | 0 | 1 | 1 | 0 |
| Sexual minority | 14.0  (8.0, 24.5) | 14.0  (5.0, 22.0) | 17.0  (8.5, 25.0) | 15.0  (9.0, 24.0) | 12.0  (6.5, 20.0) | 20.5  (13.5, 27.5) |
| Straight or heterosexual | 13.0  (9.0, 19.0) | 13.0  (8.0, 19.0) | 14.0  (9.0, 19.0) | 14.5  (8.0, 20.0) | 13.0  (8.0, 19.2) | 16.0  (10.0, 20.0) |
| **Employment status** |  |  |  |  |  |  |
| Missing (n) | 7 | 4 | 3 | 7 | 4 | 3 |
| Employed, full time | 13.0  (8.0, 19.0) | 13.0  (8.0, 19.0) | 13.0  (9.0, 19.0) | 14.0  (9.0, 20.0) | 12.0  (6.0, 18.0) | 16.5  (10.8, 20.2) |
| Employed, part time | 13.0  (9.0, 19.0) | 11.5  (8.8, 17.2) | 15.0  (9.5, 20.5) | 14.0  (7.5, 17.0) | 10.0  (7.0, 17.5) | 15.5  (12.2, 16.2) |
| Not employed | 17.5  (9.5, 24.5) | 20.0  (9.5, 25.0) | 16.0  (12.0, 22.0) | 19.0  (15.0, 23.0) | 18.0  (15.0, 21.0) | 22.5  (16.8, 23.8) |
| Primary caretaker at home, not employed outside of the home | 14.0  (6.8, 18.0) | 14.0  (6.8, 18.2) | 14.0  (9.8, 18.0) | 13.0  (8.0, 20.0) | 13.0  (8.0, 20.0) | 13.5  (8.0, 18.2) |
| **Education** |  |  |  |  |  |  |
| Missing (n) | 1 | 1 | 0 | 1 | 1 | 0 |
| College degree | 13.0  (8.8, 18.0) | 11.0  (7.2, 15.8) | 13.5  (9.2, 19.0) | 14.0  (8.2, 19.0) | 12.0  (5.0, 18.5) | 16.0  (10.0, 19.5) |
| Graduate or postgraduate degree | 12.0  (8.0, 19.0) | 11.5  (8.0, 18.0) | 13.0  (8.0, 20.0) | 15.0  (8.0, 19.0) | 12.0  (6.0, 18.0) | 17.0  (11.5, 22.5) |
| High school or GED | 16.0  (11.8, 22.5) | 16.0  (11.0, 20.0) | 16.0  (13.5, 23.5) | 17.0  (10.0, 23.5) | 18.0  (9.0, 23.0) | 16.0  (15.0, 24.5) |
| Some college or technical school | 16.0  (11.0, 21.0) | 19.5  (9.0, 22.8) | 16.0  (11.0, 19.0) | 14.0  (9.8, 22.0) | 11.5  (8.2, 20.0) | 15.5  (13.0, 22.0) |
| **Health insurance** |  |  |  |  |  |  |
| Missing (n) | 3 | 3 | 0 | 3 | 3 | 0 |
| Do not have health insurance | 18.0  (13.0, 18.0) | 11.5  (8.2, 14.8) | 18.0  (15.5, 19.5) | 14.0  (11.5, 19.0) | 16.5  (12.8, 20.2) | 14.0  (14.0, 14.0) |
| Medicaid, Medicare, or other government assistance plan | 16.0  (11.0, 23.0) | 16.0  (11.2, 22.8) | 16.0  (10.5, 23.0) | 15.0  (10.0, 22.0) | 12.0  (8.0, 20.0) | 16.0  (10.8, 25.2) |
| Private insurance | 13.0  (9.0, 19.0) | 13.0  (8.0, 19.0) | 13.0  (9.0, 19.0) | 15.0  (8.0, 20.0) | 13.0  (6.5, 19.0) | 16.0  (10.2, 21.8) |
| TRICARE, VA, or other military health care | 7.0  (5.5, 11.5) | 8.0  (7.0, 12.8) | 6.0  (5.0, 7.0) | 12.0  (4.5, 14.5) | 10.5  (6.0, 14.2) | 13.0  (4.0, 14.0) |
| **Neighborhood atlas** |  |  |  |  |  |  |
| Missing (n) | 3 | 2 | 1 | 3 | 2 | 1 |
| 25 <= ADI_NATRANK < 50 | 13.0  (8.0, 18.0) | 12.5  (7.0, 18.0) | 15.0  (11.0, 19.2) | 16.0  (9.0, 21.0) | 12.0  (8.0, 18.0) | 17.0  (12.0, 22.0) |
| 50 <= ADI_NATRANK < 75 | 15.0  (11.0, 20.0) | 15.0  (11.0, 18.8) | 16.0  (9.5, 22.2) | 15.0  (8.5, 20.0) | 13.0  (6.0, 21.0) | 15.0  (10.8, 19.0) |
| ADI_NATRANK < 25 | 12.0  (7.2, 20.0) | 11.0  (8.0, 21.0) | 12.0  (7.0, 18.2) | 13.5  (7.8, 19.0) | 13.0  (8.0, 17.5) | 15.0  (7.0, 20.0) |
| ADI_NATRANK >= 75 | 14.0  (7.0, 23.0) | 22.0  (11.5, 24.5) | 13.5  (6.2, 21.0) | 15.0  (11.0, 26.0) | 15.0  (9.5, 25.0) | 15.5  (13.2, 26.8) |

**Table S7.** PSS Stratified Scores. Numbers represent median (quartile 1, quartile 3) scores unless otherwise specified whereby n = Number of Participants. Legend: W-PPMA: WoeBot for Postpartum Mood and Anxiety, n = Number of Participants, GED = General Education Degree, VA = Veterans Affairs, ADI_Natrank = Area Deprivation Index - National Rank.

|  | **Baseline** | | | **Week 8** | | |
| --- | --- | --- | --- | --- | --- | --- |
| **Variable** | **Overall** | **W-PPMA** | **Waitlist** | **Overall** | **W-PPMA** | **Waitlist** |
| **Overall** | 5.0  (2.0, 9.0) | 5.0  (2.0, 8.0) | 5.5  (2.0, 10.0) | 5.0  (2.0, 9.0) | 4.0  (2.0, 8.0) | 6.0  (2.2, 10.0) |
| Missing (n) | 4 | 3 | 1 | 44 | 23 | 21 |
| **Age category** |  |  |  |  |  |  |
| Missing | 1 | 1 | 0 | 1 | 1 | 0 |
| <35 | 5.0  (2.0, 9.0) | 5.0  (2.2, 8.0) | 6.0  (2.2, 10.0) | 5.0  (2.0, 9.0) | 4.0  (2.0, 8.0) | 6.5  (3.0, 10.0) |
| >=35 | 4.5  (2.0, 10.0) | 3.0  (1.0, 10.8) | 5.0  (2.0, 8.5) | 5.0  (1.0, 9.5) | 3.0  (1.5, 9.0) | 5.0  (1.0, 9.2) |
| **Hispanic or Latina** |  |  |  |  |  |  |
| Missing (n) | 2 | 1 | 1 | 2 | 1 | 1 |
| No | 5.0  (2.0, 9.2) | 5.0  (2.0, 9.0) | 5.0  (2.0, 10.0) | 5.0  (2.0, 9.0) | 4.0  (2.0, 8.0) | 6.0  (2.0, 10.0) |
| Yes | 5.0  (2.2, 8.0) | 3.0  (1.5, 6.0) | 7.0  (5.0, 10.0) | 5.0  (2.5, 9.0) | 5.0  (1.0, 12.0) | 5.0  (3.5, 8.8) |
| **Racial background** |  |  |  |  |  |  |
| Missing (n) | 3 | 0 | 3 | 3 | 0 | 3 |
| Asian | 5.5  (2.5, 8.0) | 6.0  (5.0, 8.0) | 3.0  (2.0, 5.5) | 4.5  (1.8, 9.0) | 8.0  (7.0, 12.0) | 2.0  (1.5, 2.0) |
| Black/African American/African | 9.0  (2.5, 12.0) | 7.0  (2.5, 10.5) | 10.0  (5.8, 13.8) | 8.0  (3.8, 12.0) | 5.5  (2.0, 10.5) | 9.5  (4.2, 12.8) |
| Hispanic/Latina/Spanish | 5.0  (2.5, 10.0) | 5.0  (2.5, 7.0) | 10.0  (7.5, 11.5) | 6.0  (1.8, 12.8) | 7.0  (2.5, 13.0) | 5.0  (2.5, 6.5) |
| Multiple | 4.0  (2.0, 9.0) | 3.0  (1.0, 7.5) | 7.0  (4.5, 10.5) | 4.0  (2.0, 8.0) | 3.0  (1.2, 7.5) | 5.0  (4.0, 8.5) |
| White | 5.0  (2.0, 8.0) | 5.0  (2.0, 8.0) | 5.0  (2.0, 8.0) | 5.0  (2.0, 9.0) | 3.0  (2.0, 8.0) | 7.0  (2.0, 10.0) |
| **Marital status** |  |  |  |  |  |  |
| Missing (n) | 1 | 1 | 0 | 1 | 1 | 0 |
| Married/Partnered/Cohabitating | 5.0  (2.0, 8.0) | 4.0  (2.0, 8.0) | 5.0  (2.0, 9.2) | 5.0  (2.0, 9.0) | 4.0  (2.0, 8.0) | 5.0  (2.0, 10.0) |
| Single/Separated/Divorced | 8.0  (2.0, 12.0) | 7.0  (2.0, 13.0) | 9.5  (2.8, 11.5) | 7.0  (3.0, 10.5) | 3.0  (2.2, 8.8) | 8.0  (6.0, 13.0) |
| **Sexual Orientation** |  |  |  |  |  |  |
| Missing (n) | 1 | 1 | 0 | 1 | 1 | 0 |
| Sexual minority | 6.0  (3.0, 12.0) | 6.0  (3.0, 11.0) | 7.0  (3.5, 12.5) | 7.0  (3.0, 10.0) | 6.5  (3.0, 8.0) | 8.5  (4.2, 11.5) |
| Straight or heterosexual | 5.0  (2.0, 9.0) | 4.0  (2.0, 8.0) | 5.0  (2.0, 9.5) | 5.0  (2.0, 9.0) | 4.0  (2.0, 9.0) | 5.5  (2.0, 9.0) |
| **Employment status** |  |  |  |  |  |  |
| Missing (n) | 7 | 4 | 3 | 7 | 4 | 3 |
| Employed, full time | 5.0  (3.0, 9.0) | 5.0  (2.2, 8.0) | 6.0  (3.0, 9.0) | 5.0  (2.0, 9.0) | 3.0  (2.0, 8.2) | 7.0  (3.0, 10.0) |
| Employed, part time | 3.0  (2.0, 9.5) | 3.0  (2.0, 5.5) | 4.0  (1.0, 14.0) | 2.0  (1.0, 5.5) | 3.0  (1.5, 5.5) | 1.0  (0.0, 4.8) |
| Not employed | 6.0  (1.5, 12.5) | 4.5  (0.8, 14.8) | 7.0  (5.0, 10.0) | 8.0  (4.0, 12.0) | 4.0  (2.5, 10.5) | 9.0  (8.2, 12.0) |
| Primary caretaker at home, not employed outside of the home | 4.0  (1.8, 9.0) | 5.0  (1.8, 8.2) | 4.0  (1.8, 10.8) | 4.0  (1.5, 8.0) | 4.0  (2.0, 8.0) | 3.5  (1.0, 7.8) |
| **Education** |  |  |  |  |  |  |
| Missing (n) | 1 | 1 | 0 | 1 | 1 | 0 |
| College degree | 5.0  (2.0, 7.0) | 4.0  (2.0, 6.0) | 6.0  (2.0, 8.8) | 4.0  (2.0, 8.0) | 3.0  (1.2, 7.0) | 7.0  (2.0, 9.0) |
| Graduate or postgraduate degree | 4.0  (2.0, 8.0) | 4.0  (2.0, 9.0) | 4.0  (3.0, 8.0) | 5.0  (2.0, 9.0) | 4.0  (2.0, 8.0) | 5.0  (3.0, 9.5) |
| High school or GED | 6.0  (0.8, 13.0) | 6.0  (0.0, 11.0) | 11.0  (5.5, 14.5) | 7.5  (4.2, 11.0) | 7.5  (3.0, 12.2) | 8.5  6.2, 10.8) |
| Some college or technical school | 7.0  (3.0, 11.2) | 7.0  (3.2, 10.0) | 7.5  (3.2, 13.0) | 4.5  (2.0, 11.2) | 3.5  (2.2, 10.5) | 6.5  (1.5, 11.2) |
| **Health insurance** |  |  |  |  |  |  |
| Missing (n) | 3 | 3 | 0 | 3 | 3 | 0 |
| Do not have health insurance | 10.0  (5.0, 11.0) | 8.0  (6.5, 9.5) | 10.0  (5.0, 12.5) | 6.0  (3.0, 8.5) | 8.5  (7.2, 9.8) | 0.0  (0.0, 0.0) |
| Medicaid, Medicare, or other government assistance plan | 5.5  (2.0, 12.8) | 5.5  (2.0, 10.0) | 5.5  (2.0, 13.0) | 6.0  (3.0, 9.0) | 5.0  (3.0, 9.0) | 7.0  (4.0, 9.8) |
| Private insurance | 5.0  (3.0, 8.0) | 5.0  (2.8, 8.0) | 6.0  (3.0, 8.8) | 5.0  (2.0, 9.0) | 3.5  (1.8, 8.0) | 5.5  (2.2, 10.0) |
| TRICARE, VA, or other military health care | 2.0  (2.0, 6.5) | 2.0  (1.5, 7.0) | 2.0  (2.0, 3.0) | 2.0  (1.0, 5.0) | 2.0  (1.2, 2.0) | 3.0  (1.0, 7.0) |
| **Neighborhood atlas** |  |  |  |  |  |  |
| Missing (n) | 3 | 2 | 1 | 3 | 2 | 1 |
| 25 <= ADI_NATRANK < 50 | 5.0  (2.0, 7.0) | 4.5  (2.2, 7.0) | 5.0  (2.0, 9.0) | 5.0  (2.0, 9.0) | 3.0  (2.0, 8.0) | 5.0  (3.0, 9.0) |
| 50 <= ADI_NATRANK < 75 | 6.0  (3.0, 11.0) | 5.0  (2.8, 9.2) | 6.5  (4.0, 12.2) | 7.0  (2.0, 9.8) | 5.5  (2.0, 9.0) | 7.0  (3.0, 10.2) |
| ADI_NATRANK < 25 | 4.0  (2.0, 8.0) | 4.0  (1.0, 8.8) | 4.0  (2.0, 8.0) | 4.5  (1.0, 8.2) | 3.0  (1.0, 7.5) | 5.0  (2.0, 10.0) |
| ADI_NATRANK >= 75 | 7.0  (2.0, 13.0) | 8.0  (4.5, 15.5) | 4.0  (1.2, 10.8) | 4.0  (1.0, 12.0) | 4.0  (3.5, 14.0) | 2.0  (0.0, 10.5) |

**Table S8.** EPDS Stratified Scores. Numbers represent median (quartile 1, quartile 3) scores unless otherwise specified whereby n = Number of Participants. Legend: W-PPMA: WoeBot for Postpartum Mood and Anxiety, n = Number of Participants, GED = General Education Degree, VA = Veterans Affairs, ADI_Natrank = Area Deprivation Index - National Rank.

|  | **Baseline** | | | **Week 8** | | |
| --- | --- | --- | --- | --- | --- | --- |
| **Variable** | **Overall** | **W-PPMA** | **Waitlist** | **Overall** | **W-PPMA** | **Waitlist** |
| **Overall** | 4.0  (2.0, 7.0) | 4.0  (2.0, 7.0) | 5.0  (3.0, 7.0) | 5.0  (2.0, 8.0) | 4.0  (1.0, 7.0) | 5.0  (2.0, 8.0) |
| Missing | 4 | 4 | 0 | 45 | 23 | 22 |
| **Age category** |  |  |  |  |  |  |
| Missing (n) | 1 | 1 | 0 | 1 | 1 | 0 |
| <35 | 5.0  (3.0, 7.0) | 4.0  (2.0, 7.0) | 5.0  (3.0, 7.0) | 5.0  (2.0, 8.0) | 4.0  (1.0, 7.0) | 6.0  (2.0, 8.0) |
| >=35 | 3.0  (1.2, 6.0) | 3.0  (1.0, 6.8) | 3.5  (2.0, 6.0) | 3.5  (1.0, 7.0) | 3.5  (1.0, 7.5) | 3.5  (2.0, 7.0) |
| **Hispanic or Latina** |  |  |  |  |  |  |
| Missing (n) | 2 | 1 | 1 | 2 | 1 | 1 |
| No | 4.5  (2.0, 7.0) | 4.0  (2.0, 7.0) | 5.0  (3.0, 7.0) | 5.0  (2.0, 8.0) | 4.0  (1.0, 6.5) | 5.5  (2.0, 8.0) |
| Yes | 4.0  (1.2, 5.8) | 3.0  (1.0, 5.0) | 5.0  (4.0, 9.0) | 4.5  (2.0, 8.0) | 6.0  (1.2, 10.2) | 4.0  (2.2, 5.0) |
| **Racial background** |  |  |  |  |  |  |
| Missing (n) | 3 | 0 | 3 | 3 | 0 | 3 |
| Asian | 2.5  (1.8, 5.2) | 3.0  (2.0, 6.0) | 2.0  (1.5, 3.5) | 4.5  (2.5, 5.2) | 5.0  (4.0, 6.0) | 3.0  (2.0, 4.0) |
| Black/African American/African | 4.0  (3.0, 8.0) | 4.0  (2.5, 8.0) | 5.5  (3.8, 8.8) | 7.0  (4.2, 10.8) | 5.0  (3.0, 5.0) | 9.0  (7.0, 13.0) |
| Hispanic/Latina/Spanish | 4.0  (2.0, 6.0) | 4.0  (2.0, 4.5) | 7.0  (3.8, 11.0) | 5.5  (1.5, 11.0) | 8.0  (2.5, 12.0) | 3.0  (2.0, 4.0) |
| Multiple | 5.0  (2.0, 7.0) | 5.0  (2.0, 6.0) | 7.0  (4.0, 8.5) | 3.0  (1.0, 6.2) | 2.0  (1.0, 5.0) | 5.0  (3.0, 9.0) |
| White | 4.0  (2.0, 7.0) | 4.0  (2.0, 7.0) | 5.0  (3.0, 6.8) | 5.0  (2.0, 7.2) | 4.0  (1.5, 7.0) | 5.0  (2.0, 8.0) |
| **Marital status** |  |  |  |  |  |  |
| Missing (n) | 1 | 1 | 0 | 1 | 1 | 0 |
| Married/Partnered/Cohabitating | 4.0  (2.0, 7.0) | 4.0  (2.0, 7.0) | 5.0  (3.0, 7.0) | 4.5  (2.0, 7.0) | 4.0  (1.0, 7.0) | 5.0  (2.0, 8.0) |
| Single/Separated/Divorced | 4.5  (3.0, 7.0) | 4.0  (2.8, 7.5) | 6.0  (3.2, 7.0) | 6.5  (2.2, 10.0) | 4.5  (2.0, 9.2) | 8.5  (7.2, 10.2) |
| **Sexual Orientation** |  |  |  |  |  |  |
| Missing (n) | 1 | 1 | 0 | 1 | 1 | 0 |
| Sexual minority | 5.5  (3.0, 9.8) | 5.0  (3.0, 9.0) | 6.0  (3.5, 10.5) | 6.0  (3.0, 11.0) | 5.0  (2.0, 10.5) | 10.0  (6.0, 13.0) |
| Straight or heterosexual | 4.0  (2.0, 7.0) | 4.0  (2.0, 7.0) | 5.0  (2.8, 7.0) | 4.0  (2.0, 7.0) | 4.0  (1.0, 7.0) | 5.0  (2.0, 7.5) |
| **Employment status** |  |  |  |  |  |  |
| Missing (n) | 7 | 4 | 3 | 7 | 4 | 3 |
| Employed, full time | 4.0  (2.0, 7.0) | 4.0  (2.0, 7.0) | 5.0  (3.0, 6.0) | 5.0  (2.0, 7.0) | 4.0  (1.0, 7.0) | 5.0  (3.0, 7.0) |
| Employed, part time | 3.0  (2.0, 6.5) | 3.5  (2.0, 5.5) | 3.0  (1.0, 6.5) | 3.0  (1.0, 6.5) | 4.0  (1.5, 7.0) | 2.0  (1.0, 5.5) |
| Not employed | 5.0  (4.0, 10.0) | 5.0  (3.0, 14.2) | 5.0  (5.0, 6.5) | 7.0  (2.0, 10.8) | 5.0  (2.0, 14.8) | 8.5  (6.5, 9.8) |
| Primary caretaker at home, not employed outside of the home | 4.0  (2.0, 6.0) | 4.0  (0.8, 5.0) | 5.0  (3.0, 7.0) | 4.0  (2.0, 7.8) | 4.0  (2.0, 6.0) | 5.0  (2.0, 8.0) |
| **Education** |  |  |  |  |  |  |
| Missing (n) | 1 | 1 | 0 | 1 | 1 | 0 |
| College degree | 4.0  (2.0, 7.0) | 3.0  (1.0, 6.0) | 5.0  (3.0, 7.0) | 3.0  (1.0, 7.0) | 3.0  (1.0, 5.5) | 5.0  (2.0, 7.0) |
| Graduate or postgraduate degree | 4.0  (2.0, 6.0) | 3.5  (2.0, 6.0) | 4.0  (2.0, 6.5) | 5.0  (1.0, 8.0) | 4.0  (1.0, 7.2) | 6.0  (3.0, 8.5) |
| High school or GED | 5.0  (2.8, 8.5) | 4.0  (2.0, 6.0) | 8.0  (5.0, 10.5) | 6.0  (3.0, 13.8) | 5.5  (2.0, 13.2) | 7.5  (5.5, 13.2) |
| Some college or technical school | 5.0  (3.0, 8.0) | 5.5  (3.0, 8.0) | 5.0  (3.0, 6.5) | 5.0  (2.0, 8.2) | 5.0  (2.0, 6.0) | 5.0  (2.0, 8.8) |
| **Health insurance** |  |  |  |  |  |  |
| Missing (n) | 3 | 3 | 0 | 3 | 3 | 0 |
| Do not have health insurance | 5.0  (4.0, 7.0) | 5.5  (4.8, 6.2) | 5.0  (4.0, 6.0) | 6.0  (3.5, 6.0) | 6.0  (6.0, 6.0) | 1.0  (1.0, 1.0) |
| Medicaid, Medicare, or other government assistance plan | 5.0  (2.2, 8.0) | 5.0  (2.2, 7.8) | 6.5  (2.8, 9.2) | 5.0  (2.0, 9.5) | 5.0  (2.0, 6.0) | 7.0  (3.0, 10.0) |
| Private insurance | 4.0  (2.0, 6.5) | 3.5  (2.0, 7.0) | 5.0  (3.0, 6.0) | 5.0  (2.0, 8.0) | 4.0  (1.0, 8.0) | 5.0  (2.0, 8.0) |
| TRICARE, VA, or other military health care | 3.0  (1.5, 5.0) | 5.0  (2.5, 6.8) | 2.0  (1.0, 3.0) | 1.0  (1.0, 5.0) | 1.0  (1.0, 2.5) | 4.0  (1.0, 6.0) |
| **Neighborhood atlas** |  |  |  |  |  |  |
| Missing (n) | 3 | 2 | 1 | 3 | 2 | 1 |
| 25 <= ADI_NATRANK < 50 | 4.0  (2.0, 7.0) | 3.0  (2.0, 5.8) | 5.0  (2.8, 7.0) | 5.0  (2.0, 8.0) | 4.0  (1.0, 7.0) | 6.0  (2.5, 8.0) |
| 50 <= ADI_NATRANK < 75 | 5.0  (3.0, 7.0) | 5.0  (2.0, 7.0) | 5.0  (3.8, 7.0) | 5.0  (2.0, 8.0) | 5.0  (2.0, 7.5) | 5.0  (2.8, 8.2) |
| ADI_NATRANK < 25 | 4.0  (2.0, 6.0) | 4.0  (2.0, 6.8) | 4.0  (2.8, 6.0) | 4.0  (1.0, 6.5) | 3.0  (1.0, 6.5) | 5.0  (2.0, 6.0) |
| ADI_NATRANK >= 75 | 4.0  (2.0, 10.0) | 8.0  (4.0, 11.0) | 3.5  (1.2, 8.8) | 4.5  (1.0, 14.2) | 5.0  (3.0, 13.5) | 2.0  (1.0, 15.0) |

**Table S9.** GAD-7 Stratified Scores. Numbers represent median (quartile 1, quartile 3) scores unless otherwise specified whereby n = Number of Participants. Legend: W-PPMA: WoeBot for Postpartum Mood and Anxiety, n = Number of Participants, GED = General Education Degree, VA = Veterans Affairs, ADI_Natrank = Area Deprivation Index - National Rank.

|  | **Baseline** | | | **Week 8** | | |
| --- | --- | --- | --- | --- | --- | --- |
| **Variable** | **Overall** | **W-PPMA** | **Waitlist** | **Overall** | **W-PPMA** | **Waitlist** |
| **Overall** | 3.0  (1.0, 5.0) | 3.0  (1.0, 5.0) | 3.0  (1.0, 5.0) | 3.0  (1.0, 5.0) | 2.5  (1.0, 4.0) | 3.0  (1.0, 6.0) |
| Missing (n) | 8 | 6 | 2 | 43 | 22 | 21 |
| **Age category** |  |  |  |  |  |  |
| Missing (n) | 1 | 1 | 0 | 1 | 1 | 0 |
| <35 | 3.0  (1.0, 5.0) | 3.0  (1.0, 5.0) | 3.0  (1.0, 5.0) | 3.0  (1.0, 4.0) | 2.0  (1.0, 3.0) | 3.0  (1.0, 5.8) |
| >=35 | 3.0  (1.0, 5.0) | 3.0  (2.0, 5.0) | 4.0  (1.0, 5.0) | 3.0  (1.0, 6.0) | 3.0  (1.0, 6.0) | 3.5  (1.0, 6.0) |
| **Hispanic or Latina** |  |  |  |  |  |  |
| Missing (n) | 2 | 1 | 1 | 2 | 1 | 1 |
| No | 3.0  (1.0, 5.0) | 3.0  (1.0, 5.0) | 3.0  (1.0, 5.0) | 3.0  (1.0, 5.0) | 2.0  (1.0, 4.0) | 3.0  (1.0, 5.5) |
| Yes | 4.0  (2.2, 6.0) | 3.0  (2.0, 6.5) | 5.0  (3.5, 6.0) | 3.0  (2.0, 6.5) | 3.0  (2.0, 4.0) | 3.0  (2.0, 7.5) |
| **Racial background** |  |  |  |  |  |  |
| Missing (n) | 3 | 0 | 3 | 3 | 0 | 3 |
| Asian | 1.0  (0.5, 4.0) | 3.0  (1.0, 5.0) | 0.5  (0.2, 0.8) | 3.5  (2.5, 8.2) | 3.0  (3.0, 6.0) | 4.0  (2.0, 9.5) |
| Black/African American/African | 4.0  (1.0, 6.5) | 5.0  (1.0, 6.0) | 3.0  (0.8, 7.0) | 3.5  (1.5, 5.5) | 3.5  (0.8, 4.0) | 4.0  (2.2, 7.0) |
| Hispanic/Latina/Spanish | 5.0  (2.0, 6.5) | 4.0  (2.0, 6.5) | 5.5  (4.0, 7.8) | 3.0  (1.0, 9.0) | 3.0  (2.0, 9.2) | 0.0  (0.0, 4.5) |
| Multiple | 3.0  (1.0, 5.0) | 2.0  (1.0, 4.0) | 4.0  (1.5, 5.5) | 2.0  (1.0, 4.0) | 2.0  (1.2, 3.0) | 2.0  (1.5, 5.5) |
| White | 3.0  (1.0, 5.0) | 3.0  (1.0, 5.0) | 3.0  (1.0, 5.0) | 3.0  (1.0, 4.0) | 2.0  (1.0, 4.0) | 3.0  (1.0, 5.0) |
| **Marital status** |  |  |  |  |  |  |
| Missing (n) | 1 | 1 | 0 | 1 | 1 | 0 |
| Married/Partnered/Cohabitating | 3.0  (1.0, 5.0) | 3.0  (1.0, 5.0) | 3.0  (1.0, 5.0) | 3.0  (1.0, 5.0) | 2.5  (1.0, 4.0) | 3.0  (1.0, 6.0) |
| Single/Separated/Divorced | 2.0  (1.0, 5.8) | 2.5  (1.0, 5.2) | 1.5  (0.0, 5.8) | 3.0  (1.0, 4.5) | 2.5  (0.2, 3.8) | 5.0  (2.0, 7.0) |
| **Sexual Orientation** |  |  |  |  |  |  |
| Missing (n) | 1 | 1 | 0 | 1 | 1 | 0 |
| Sexual minority | 2.0  (1.0, 4.2) | 3.0  (1.0, 5.0) | 2.0  (1.0, 3.0) | 1.0  (0.0, 3.0) | 1.0  (0.0, 3.0) | 0.5  (0.0, 2.0) |
| Straight or heterosexual | 3.0  (1.0, 5.5) | 3.0  (1.0, 6.0) | 3.0  (1.0, 5.0) | 3.0  (1.0, 6.0) | 3.0  (1.0, 4.0) | 4.0  (1.0, 6.0) |
| **Employment status** |  |  |  |  |  |  |
| Missing (n) | 7 | 4 | 3 | 7 | 4 | 3 |
| Employed, full time | 3.0  (1.0, 6.0) | 3.0  (2.0, 6.0) | 3.0  (1.0, 5.0) | 3.0  (1.0, 5.0) | 3.0  (1.0, 4.0) | 3.0  (1.0, 5.2) |
| Employed, part time | 3.0  (2.0, 5.8) | 3.0  (2.5, 4.0) | 4.0  (2.0, 8.0) | 3.5  (1.0, 7.5) | 2.0  (1.0, 3.8) | 5.5  (3.0, 8.2) |
| Not employed | 2.0  (0.5, 5.0) | 3.5  (0.0, 7.5) | 2.0  (1.5, 3.0) | 3.0  (1.0, 7.0) | 3.0  (1.0, 4.5) | 5.5  (3.2, 7.0) |
| Primary caretaker at home, not employed outside of the home | 3.0  (1.0, 5.0) | 3.0  (1.0, 5.0) | 3.0  (1.0, 5.0) | 2.0  (0.0, 4.0) | 2.0  (0.0, 3.0) | 2.0  (0.0, 4.0) |
| **Education** |  |  |  |  |  |  |
| Missing (n) | 1 | 1 | 0 | 1 | 1 | 0 |
| College degree | 3.0  (1.0, 6.0) | 3.0  (1.0, 5.5) | 3.5 (1.0, 6.0) | 2.0  (1.0, 4.0) | 2.0  (0.5, 3.5) | 3.0  (1.0, 6.0) |
| Graduate or postgraduate degree | 3.0  (1.0, 5.0) | 3.0  (2.0, 5.0) | 2.0  (1.0, 5.0) | 3.0  (1.0, 5.0) | 3.0  (2.0, 4.2) | 3.0  (1.0, 5.0) |
| High school or GED | 4.5  (1.0, 6.2) | 4.0  (1.0, 6.0) | 5.0  (1.0, 6.5) | 3.0  (1.0, 6.5) | 3.0  (2.0, 7.0) | 3.0  (0.0, 6.0) |
| Some college or technical school | 3.5  (1.8, 6.2) | 4.0  (2.0, 7.0) | 3.0  (1.5, 5.5) | 2.5  (0.0, 5.0) | 1.0  (0.0, 3.5) | 4.0  (3.0, 5.8) |
| **Health insurance** |  |  |  |  |  |  |
| Missing (n) | 3 | 3 | 0 | 3 | 3 | 0 |
| Do not have health insurance | 5.0  (3.0, 7.0) | 3.5  (1.8, 5.2) | 5.0  (4.0, 6.0) | 0.0  (0.0, 5.5) | 5.5  (2.8, 8.2) | 0.0  (0.0, 0.0) |
| Medicaid, Medicare, or other government assistance plan | 4.0  (1.0, 6.0) | 4.0  (1.0, 7.0) | 3.5  (1.0, 6.0) | 3.0  (1.0, 7.0) | 2.0  (0.5, 4.0) | 4.0  (1.0, 7.0) |
| Private insurance | 3.0  (1.0, 5.0) | 3.0  (1.0, 5.0) | 3.0  (1.0, 5.0) | 3.0  (1.0, 5.0) | 3.0  (1.0, 4.0) | 3.0  (1.0, 5.0) |
| TRICARE, VA, or other military health care | 3.0  (1.0, 4.5) | 3.5  (3.0, 4.8) | 1.0  (1.0, 2.0) | 4.0  (0.5, 4.0) | 4.0  (2.5, 4.0) | 1.0  (0.0, 4.0) |
| **Neighborhood atlas** |  |  |  |  |  |  |
| Missing (n) | 3 | 2 | 1 | 3 | 2 | 1 |
| 25 <= ADI_NATRANK < 50 | 3.0  (1.0, 5.0) | 3.0  (1.0, 5.0) | 2.5  (1.0, 5.0) | 3.0  (1.0, 4.0) | 2.0  (1.0, 4.0) | 3.0  (1.0, 4.0) |
| 50 <= ADI_NATRANK < 75 | 3.5  (1.0, 6.0) | 4.0  (1.0, 6.0) | 3.0  (1.0, 7.0) | 3.0  (1.0, 6.0) | 2.0  (1.0, 3.0) | 4.0  (1.0, 6.0) |
| ADI_NATRANK < 25 | 3.0  (1.0, 5.0) | 3.0  (1.0, 5.0) | 3.0  (1.5, 5.0) | 3.0  (1.0, 4.5) | 3.0  (1.0, 4.0) | 3.0  (1.0, 5.0) |
| ADI_NATRANK >= 75 | 2.0  (1.0, 6.0) | 2.0  (0.5, 7.0) | 2.5  (1.2, 5.8) | 3.0  (1.0, 9.0) | 3.0  (1.0, 6.5) | 4.0  (1.2, 8.5) |

**Table S10.** MIB Stratified Scores. Numbers represent median (quartile 1, quartile 3) scores unless otherwise specified whereby n = Number of Participants. Legend: W-PPMA: WoeBot for Postpartum Mood and Anxiety, n = Number of Participants, GED = General Education Degree, VA = Veterans Affairs, ADI_Natrank = Area Deprivation Index - National Rank.

| **Variable** | **Day 3** | **Week 8** |
| --- | --- | --- |
| **Overall** | 42.0 (36.0, 51.0) | 48.0 (41.0, 53.0) |
| Missing (n) | 50 | 31 |
| **Age category** |  |  |
| Missing (n) | 1 | 1 |
| <35 | 44.0 (37.0, 51.0) | 50.0 (41.2, 53.8) |
| >=35 | 37.5 (35.0, 47.2) | 47.0 (40.2, 51.8) |
| **Hispanic or Latina** |  |  |
| Missing (n) | 1 | 1 |
| No | 41.0 (36.0, 50.0) | 48.0 (42.0, 53.0) |
| Yes | 49.0 (37.0, 54.0) | 45.5 (37.0, 54.2) |
| **Racial background** |  |  |
| Missing (n) | 0 | 0 |
| Asian | 37.5 (36.5, 41.2) | 45.0 (36.0, 48.0) |
| Black/African American/African | 50.5 (40.5, 55.2) | 48.0 (46.0, 50.0) |
| Hispanic/Latina/Spanish | 49.0 (45.0, 51.0) | 51.0 (37.5, 53.8) |
| Multiple | 48.0 (36.5, 54.5) | 51.0 (42.2, 55.2) |
| White | 41.0 (36.0, 49.0) | 47.0 (41.0, 53.0) |
| **Marital status** |  |  |
| Missing (n) | 1 | 1 |
| Married/Partnered/Cohabitating | 43.0 (36.0, 51.0) | 48.0 (40.5, 53.0) |
| Single/Separated/Divorced | 40.0 (38.2, 51.2) | 50.0 (43.2, 55.8) |
| **Sexual Orientation** |  |  |
| Missing (n) | 1 | 1 |
| Sexual minority | 41.0 (38.0, 49.0) | 43.0 (37.0, 49.0) |
| Straight or heterosexual | 43.0 (36.0, 51.0) | 49.0 (41.0, 53.0) |
| **Employment status** |  |  |
| Missing (n) | 4 | 4 |
| Employed, full time | 43.5 (36.0, 51.0) | 47.0 (41.0, 53.0) |
| Employed, part time | 39.5 (33.8, 43.8) | 49.5 (38.2, 55.8) |
| Not employed | 38.0 (34.5, 40.5) | 50.5 (39.0, 54.5) |
| Primary caretaker at home, not employed outside of the home | 47.5 (37.8, 51.0) | 50.0 (43.0, 56.0) |
| **Education** |  |  |
| Missing (n) | 1 | 1 |
| College degree | 42.0 (37.0, 50.5) | 47.0 (40.0, 51.0) |
| Graduate or postgraduate degree | 40.5 (35.0, 49.2) | 48.0 (42.0, 53.0) |
| High school or GED | 40.0 (36.5, 49.5) | 51.0 (42.8, 55.5) |
| Some college or technical school | 48.5 (39.2, 52.2) | 49.0 (37.0, 55.0) |
| **Health insurance** |  |  |
| Missing (n) | 3 | 3 |
| Do not have health insurance | N/A | 34.5 (26.2, 42.8) |
| Medicaid, Medicare, or other government assistance plan | 40.0 (36.2, 52.8) | 50.0 (37.0, 56.0) |
| Private insurance | 42.5 (36.2, 49.8) | 48.0 (43.0, 53.0) |
| TRICARE, VA, or other military health care | 42.0 (33.0, 50.5) | 52.0 (47.0, 58.0) |
| **Neighborhood atlas** |  |  |
| Missing (n) | 2 | 2 |
| 25 <= ADI_NATRANK < 50 | 42.5 (37.2, 51.0) | 48.5 (43.0, 54.2) |
| 50 <= ADI_NATRANK < 75 | 41.5 (36.8, 49.8) | 49.5 (40.2, 53.8) |
| ADI_NATRANK < 25 | 39.0 (36.0, 50.0) | 47.0 (36.8, 52.0) |
| ADI_NATRANK >= 75 | 52.0 (37.0, 53.5) | 55.0 (47.0, 55.5) |

**Table S11.** WAI-SR Stratified Scores. Numbers represent median (quartile 1, quartile 3) scores unless otherwise specified whereby n = Number of Participants. Legend: W-PPMA: WoeBot for Postpartum Mood and Anxiety, n = Number of Participants, GED = General Education Degree, VA = Veterans Affairs, ADI_Natrank = Area Deprivation Index - National Rank.
